# Molecular phylogeny reveals food plasticity in the evolution of true ladybird beetles (Coleoptera: Coccinellidae: Coccinellini)

**DOI:** 10.1186/s12862-017-1002-3

**Published:** 2017-06-26

**Authors:** Hermes E. Escalona, Andreas Zwick, Hao-Sen Li, Jiahui Li, Xingmin Wang, Hong Pang, Diana Hartley, Lars S. Jermiin, Oldřich Nedvěd, Bernhard Misof, Oliver Niehuis, Adam Ślipiński, Wioletta Tomaszewska

**Affiliations:** 1 Centre for Molecular Biodiversity Research (ZMB), Museum Alexander Koenig, Adenauerallee, 53113 Bonn, Germany; 2 Australian National Insect Collection, CSIRO, GPO Box 1700, Canberra, ACT 2601 Australia; 3 State Key Laboratory of Biocontrol, Key Laboratory of Biodiversity Dynamics and Conservation of Guangdong Higher Education Institute, College of Ecology and Evolution, Sun Yat-Sen University, Guangzhou, 510275 China; 4 College of Environment and Plant Protection, Hainan University, No. 58 Renmin Avenue, Haikou, 570228 China; 5 Key Laboratory of Bio-Pesticide Innovation and Application, Guangdong Province, Guangzhou, China; 6 Centre for Biodiversity Analysis, Australian National University, ACT, Acton, 2601 Australia; 7 Institute of Entomology, Biology Centre, Branišovská 31, -37005 České Budějovice, CZ Czech Republic; 8 University of South Bohemia, Branišovská, 31 České Budějovice, Czech Republic; 9 Department of Evolutionary Biology and Ecology, Institute of Biology I (Zoology) Albert Ludwig University of Freiburg, Hauptstr. 1, 79104 Freiburg, Germany; 10 Museum and Institute of Zoology, Polish Academy of Sciences, Wilcza 64, 00-679 Warszawa, Poland

**Keywords:** Coccinelloidea, Ladybugs, Diet shifts, Evolution, Feeding strategies, Food preferences, Taxonomy

## Abstract

**Background:**

The tribe Coccinellini is a group of relatively large ladybird beetles that exhibits remarkable morphological and biological diversity. Many species are aphidophagous, feeding as larvae and adults on aphids, but some species also feed on other hemipterous insects (i.e., heteropterans, psyllids, whiteflies), beetle and moth larvae, pollen, fungal spores, and even plant tissue. Several species are biological control agents or widespread invasive species (e.g., *Harmonia axyridis* (Pallas)). Despite the ecological importance of this tribe, relatively little is known about the phylogenetic relationships within it. The generic concepts within the tribe Coccinellini are unstable and do not reflect a natural classification, being largely based on regional revisions. This impedes the phylogenetic study of important traits of Coccinellidae at a global scale (e.g. the evolution of food preferences and biogeography).

**Results:**

We present the most comprehensive phylogenetic analysis of Coccinellini to date, based on three nuclear and one mitochondrial gene sequences of 38 taxa, which represent all major Coccinellini lineages. The phylogenetic reconstruction supports the monophyly of Coccinellini and its sister group relationship to Chilocorini. Within Coccinellini, three major clades were recovered that do not correspond to any previously recognised divisions, questioning the traditional differentiation between Halyziini, Discotomini, Tytthaspidini, and Singhikaliini. Ancestral state reconstructions of food preferences and morphological characters support the idea of aphidophagy being the ancestral state in Coccinellini. This indicates a transition from putative obligate scale feeders, as seen in the closely related Chilocorini, to more agile general predators.

**Conclusions:**

Our results suggest that the classification of Coccinellini has been misled by convergence in morphological traits. The evolutionary history of Coccinellini has been very dynamic in respect to changes in host preferences, involving multiple independent host switches from different insect orders to fungal spores and plants tissues. General predation on ephemeral aphids might have created an opportunity to easily adapt to mixed or specialised diets (e.g. obligate mycophagy, herbivory, predation on various hemipteroids or larvae of leaf beetles (Chrysomelidae)). The generally long-lived adults of Coccinellini can consume pollen and floral nectars, thereby surviving periods of low prey frequency. This capacity might have played a central role in the diversification history of Coccinellini.

**Electronic supplementary material:**

The online version of this article (doi:10.1186/s12862-017-1002-3) contains supplementary material, which is available to authorized users.

## Background

Ladybirds (Coccinellidae) are a well-defined monophyletic group of small to medium sized beetles of the superfamily Coccinelloidea, the superfamily formerly known as the Cerylonid Series within the superfamily Cucujoidea [1–3]. The relationships between the currently recognized 15 families of Coccinelloidea are not well understood, but comprehensive molecular phylogenetic analyses of Coccinelloidea [2] suggested that Eupsilobiidae, a mycophagous group of small brown beetles, previously included as a subfamily of Endomychidae [4, 5], are the sister group of Coccinellidae. Coccinellidae, which comprises 360 genera and about 6000 species world-wide, is by far the largest family of coccinelloid beetles and, with the notable exception of the parasitic Bothrideridae, the only predominantly predatory lineage of Coccinelloidea. The ancestor of Coccinellidae presumably lived in the Jurassic (~ 150 Mya [6]) and even a Permian-Triassic origin of Coccinelloidea has been suggested [7]. The development of a predatory life style in the ancestor of Coccinellidae, was possibly a relevant event for the evolutionary history of this beetle lineage, with herbivory, sporophagy and pollenophagy being derived from this predatory mode of life.

Most of the traditional classifications of Coccinellidae [8–10] recognize six or seven subfamilies (i.e., Chilocorinae, Coccidulinae, Coccinellinae, Epilachninae, Scymninae, Sticholotidinae, and sometimes Ortaliinae, each with numerous tribes). The foundation of this system was developed by Sasaji [11, 12] based on comparative morphological analyses of adults and larvae from species of the Palaearctic Region, mostly Japan. Kovář [9] presented a major modification of Sasaji’s classification on a global scale, recognizing seven subfamilies and 38 tribes. The classifications proposed by Sasaji [11] and Kovář [9] were found to be largely artificial and phylogenetically unacceptable by Ślipiński [13], who argued for a basal split of Coccinellidae into two subfamilies, Microweiseinae and Coccinellinae, with the latter containing most of the tribes, including Coccinellini. Six subsequent papers on the molecular phylogeny of the family Coccinellidae [14–17] and Cucujoidea [2, 3] corroborated the monophyly of the family and of the two subfamilies recognized by Ślipiński [13]. They also provided strong evidence for the monophyly of Coccinellini. Based on results of phylogenetic analyses of molecular data and a combination of molecular and morphological data from Coccinellidae, Ślipiński and Tomaszewska [18] and Seago et al. [17] formalized the taxonomic status of Coccinellini as a tribe within the broadly defined Coccinellinae.

Coccinellini, commonly referred to as ‘true ladybirds’, comprises 90 genera and over 1000 species world-wide. The tribe includes many charismatic and easily recognised beetles that are often seen on aphid-infested trees and shrubs in the natural and urban landscapes. It is also one of the most frequently studied groups of beetles, the subject of thousands of peer-reviewed scientific papers on biology, genetics, colour polymorphism, physiology and biological control, summarized in various influential books [19–22].

**Fig. 1:**
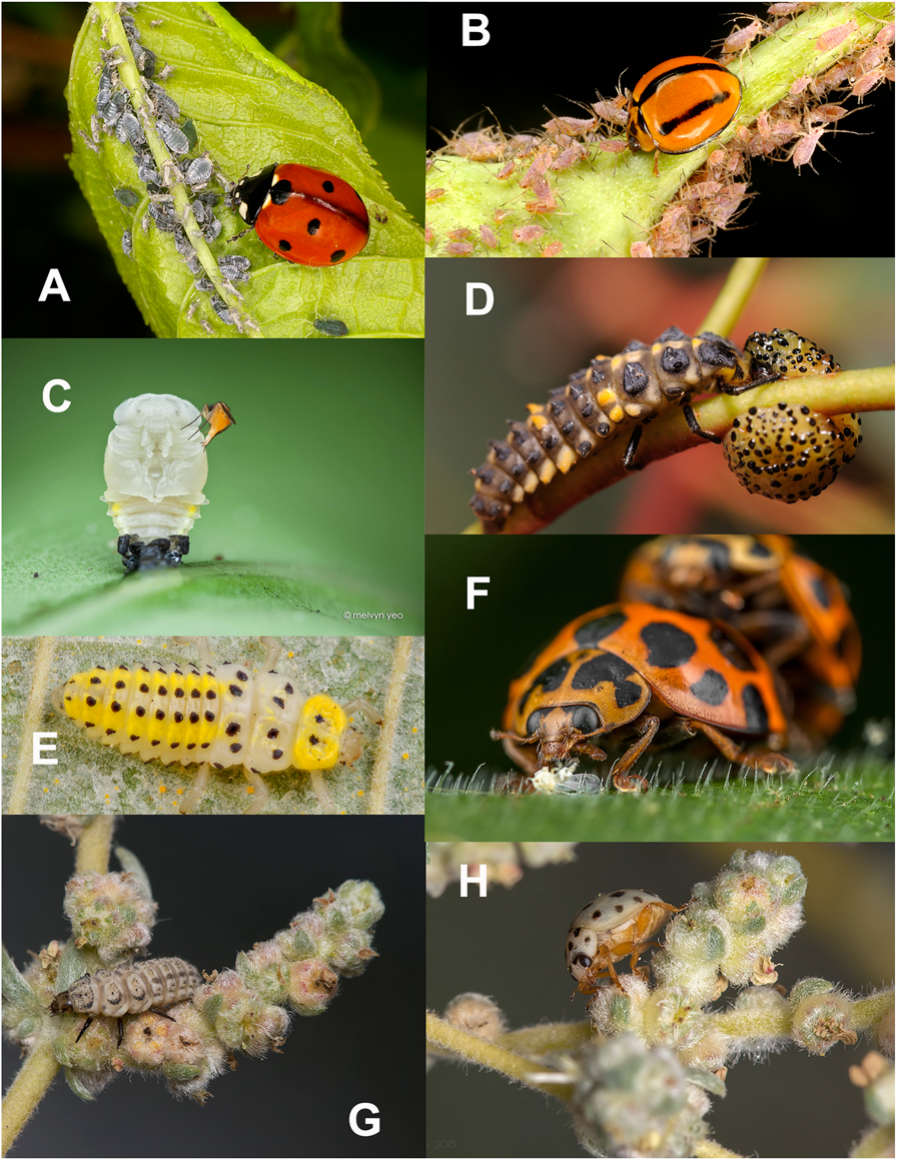
Representative spectrum of Coccinellini morphologies and feeding habits: **a** *Coccinella septempunctata*, adult feeding on aphids; **b** *Coelophora variegata*, adult feeding on aphids; **c** *Heteroneda reticulata*, pupa being parasitized by a phorid fly; **d** *Cleobora mellyi*, larva feeding on larva of *Paropsis charybdis* (Chrysomelidae); **e** *Halyzia sedecimguttata*, larva feeding on mildew; **f** *Harmonia conformis*, adult feeding on psyllids; **g**, **h** *Bulaea lichatschovi*, larva and adult, feeding on leaves and buds of *Bassia prostrata*. Photographs credits: **a**, **b** Paul Zborowski; **c** Melvyn Yeo; **d** Andrew Bonnitcha; **e** Gilles San Martin; **f** Nick Monaghan; **g**, **h** Maxim Gulyaev

**Fig. 2:**
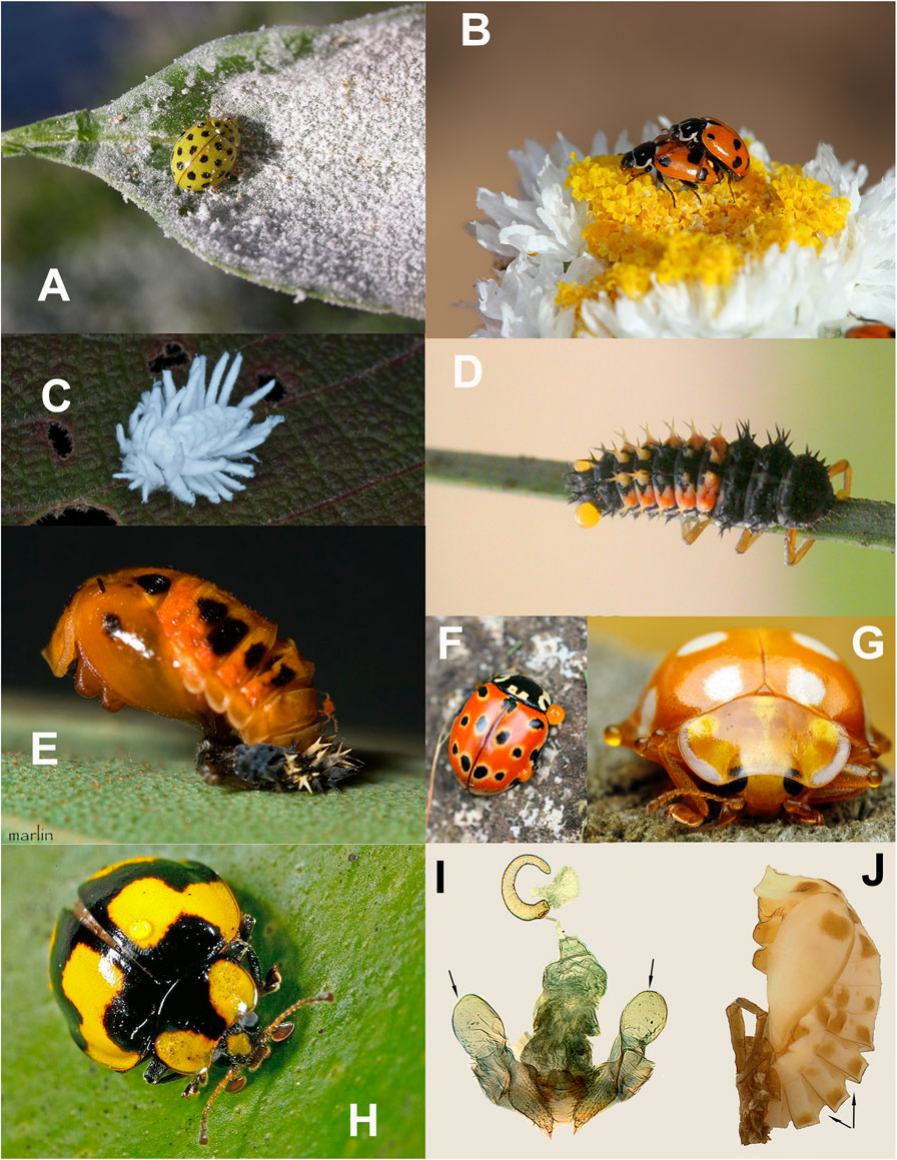
Representative spectrum of Coccinellidae morphologies and feeding habits. **a** *Psyllobora vigintiduopunctata*, adult feeding on mildew; **b** *Hippodamia variegata*, adults feeding on pollen; **c** *Scymnus* sp., larva with dense waxy covering; **d** *Harmonia axyridis*, larva showing droplets of haemolymph at abdominal segments; **e** *Harmonia axyridis*, pupa with nymph of parasitic mite; **f** *Anatis ocellata*, adult with excreted droplets of haemolymph; **g** *Halyzia sedecimguttata*, adult with excreted haemolymph droplets on legs; **h** *Illeis galbula*, adult, showing strongly expanded terminal maxillary palpomere; **i** *Phrynocaria astrolabiana*, female terminalia showing glands (indicated by *arrows*); **j** *Archegleis kingi*, pupa lateral showing gin traps between abdominal tergites (indicated by *arrows*). Photographs credits: **a** Jelle Devalez; **b** Nick Monaghan; **c** Paul Zborowski; **d** Gilles San Martin; **e** Bruce Marlin; **f** Remy Ware; **g** John Jeffery; **h** Steve Axford

Coccinellini are generally viewed as predators of aphids, but their diet is much more diverse and often includes other hemipterous insects (i.e., heteropterans, psyllids), beetle and moth larvae, pollen, fungal spores, and even plant tissues. Coccinellini display extraordinary morphological diversity in all life stages and are among the most conspicuously and attractively coloured beetles, often bearing strikingly red or yellow elytra, with contrasting black spots, stripes, or fasciae (Figs. 1 and 2). These vivid colours are aposematic, warning predators that these beetles are distasteful and produce noxious or poisonous alkaloids [23] excreted as droplets of fluid during a ‘reflex bleeding’ behaviour. Many species of Coccinellini are also of great economic importance as biological control agents or unwanted invaders on a scale of entire continents (e.g., multicoloured Asian ladybird beetle, *Harmonia axyridis* [24]).

Surprisingly, relatively little is known about the phylogenetic relationships and the evolutionary history of this ecologically important and species-rich beetle lineage. The phylogeny of the tribe has not been studied in detail and its subordinated taxonomic classification is largely regional and non-phylogenetic, impeding comparative analyses of important features of coccinellid evolution, such as host preferences, on a global scale. So far, published research on the evolutionary history of Coccinellidae has focussed on the phylogeny of the entire family and included only a very limited set of Coccinellini. The study by Magro et al. [15] included more species and genera of Coccinellini (i.e., 32 species, 15 genera) than any other investigation, but the authors’ taxon sampling was heavily focused on European species. Their data set differed from a smaller set of Asian taxa (24 species, 15 genera) analysed by Aruggoda et al. [16] and a similar sized but more global data set (23 species, 16 genera) by Robertson et al. [2]. In addition to the taxonomically different data sets, the molecular hypotheses put forth in the cited papers had very weak support especially at deeper nodes within Coccinellini, and each study recovered incongruent relationships among the genera. More comprehensive morphological and molecular research is required to improve the global classification of Coccinellini and to establish a reliable generic classification for this tribe.

Here we present molecular phylogenetic analyses based on a world-wide and taxonomically broad sampling of Coccinellini, representing all major lineages and analysing the phylogenetic signal of four genes (one mitochondrial and three nuclear) using Bayesian and Maximum-Likelihood (ML) phylogenetic approaches. The aims of our study are to: (1) assess the monophyly of Coccinellini; (2) generate the first comprehensive phylogenetic hypothesis about generic relationships within the tribe Coccinellini; (3) test if some formerly recognised tribes of Coccinellini (i.e., Discotomini, Halyziini, Singhikaliini, and Tytthaspidini) merit recognition as subtribes; and (4) reconstruct the evolution of selected morphological characters and of food preferences within Coccinellini.

## Methods

### Taxon sampling and morphology

We analysed 38 species of Coccinellini belonging to 32 of 90 genera. They represent all previously proposed tribes currently included in Coccinellini (i.e., Coccinellini – 23 of 67 genera, Discotomini – 1 of 5 genera, Halyziini – 3 of 8 genera, Singhikaliini – 1 genus (monotypic tribe), and Tytthaspidini (=Bulaeini) – 4 of 9 genera) and 14 outgroup species, representing a variety of coccinellid subfamilies and tribes, and two species of Corylophidae. Our taxon sampling was not designed to assess relationships within the family Coccinellidae, but was aimed at inferring the relationships within the tribe Coccinellini and tracing the evolution of morphological traits and food preferences. We selected species with known biology and food preferences, if tissue samples containing DNA were available to us. The biology of *Seladia beltiana* Gorham (former Discotomini) and *Singhikalia duodecimguttata* Xiao (former Singhikaliini) is unknown, but the examination of gut contents of two specimens of *Seladia* sp. revealed abundant fungal spores, suggesting that this species may be fungivorous (A. Ślipiński, personal observation). Gut contents of *Singhikalia duodecimguttata* Xiao from China and *S. latemarginata* (Bielawski) from Papua New Guinea showed a mixture of unrecognizable cuticular pieces and fungal conidia (A. Ślipiński, personal observation), which indicates a mixed or fungal diet.

We compiled a data matrix with essential information on food preference and the state of six morphological characters of adults and immatures for each species in our study (Additional file 1: Table S3). Morphological characters selected (adult pubescence, female colleterial glands [13], larval dorsal gland openings, larval wax secretions, and pupal gin traps) have been used as diagnostic characters for Coccinellini [8, 10, 12, 13] or (mandible type) used in discussions about the food preferred by adult beetles [25, 26] but none of these have been phylogenetically tested. Morphological characters were obtained from voucher specimens at the Australian National Insect Collection (CSIRO) and the literature [8, 9, 11]. The primary food preference (essential food source) of each species was established from the dissected guts of several representatives of each species and from the literature [8, 14, 21, 27–32].

### DNA sequencing of target genes

DNA was extracted from ethanol preserved specimens following the standard protocol for animal tissues of the Qiagen DNeasy Blood and Tissue kit. Generally, one specimen per species was used for the extraction. Four nuclear and one mitochondrial gene fragments were amplified by PCR (i.e., two sections of carbamoylphosphate synthetase / aspartate transcarbamylase / dihydroorotase (CAD: CADMC and CADXM), topoisomerase I (TOPO), wingless (WGL), and cytochrome oxidase I (COI). These genes contrary to the widely used ribosomal genes (e.g. 18S, 28S) can be aligned with more accuracy. The amplification strategy [33], using degenerate primers with M13 (−21) / M13REV tails attached to the 5′ ends of the forward and the reverse primer, respectively. The primers had either been published previously [34] or were developed by us in context of the present study (CADXM2; Additional file 2: Table S1). Depending on the PCR yield, PCR products were either sequenced directly or re-amplified in a second and/or third PCR with hemi-nested and / or M13 primers. Initial PCRs were performed in 50-μL reaction volumes (32.8 μL of water, 5 μL of 10× buffer, 4 μL of 25 mM MgCl_2_, 2 μL of 10 mM dNTP mix, 2 μL of each 10 mM forward and reverse primer, 0.2 μL of 5 U/μL KAPA taq polymerase, 2 μL of template DNA) and a touch-down temperature profile that stepped from 55 °C down to 45 °C for conveniently amplifying with all primer pairs, irrespective of their specific binding temperature, 25 cycles with 94 °C for 30 s., 55 °C [−0.4 °C each cycle] for 30 s., and 72 °C, for 60 s. [+ 2 s. each cycle], followed by 13 cycles with 94 °C for 30 s., 45 °C for 30 s, 72 °C for 120 s [+ 3 s. each cycle], followed by 72 °C for 600 s. [35]. Reamplifications also used 50-μl PCR reactions, but a simplified three-step hot-start temperature profile (22 cycles with 94 °C for 30 s., 50 °C for 30 s., and 72 °C for 60 s. [+ 2 s. each cycle], followed by 72 °C for 600 s.). All PCR products were bidirectionally sequenced using Sanger sequencing technology provided by LGC Genomics (Berlin, Germany). All raw reads were assembled with Geneious (v9.1.5; Biomatters, New Zealand [36]) and manually checked for sequencing errors, ambiguities and if necessary, manually edited.

### Phylogenetic analyses

The coding DNA sequence of each gene was translated to the corresponding amino-acid sequence with the software Virtual Ribosome (version 2.0; [37]). The amino-acid sequences of each gene (CADMC, CADXM, TOPO, WGL, COI) were aligned using MAFFT (version 7.164b; [38]) and the original nucleotide sequences were mapped onto the alignments of amino-acid with a Perl script to generate a codon-based alignment of the nucleotide sequences (available upon request to AZ). The nucleotide and amino-acid multiple sequence alignments (MSAs) were visually inspected, and ambiguously aligned or gapped areas were excluded from downstream analyses (i.e., 194 of 3485 sites in the MSAs). All nucleotide sequences were queried against GenBank (NCBI [39]) using the software BLAST+ [40] to check for potential contaminations (e.g., gut content, fungi). We also inferred a neighbour-joining tree (PAUP*4.0b10, Linux, Sinauer Associates, MA, USA; [41]), from the nucleotide sequence of each gene fragment to check for potential cross-contaminations and sample swapping (the results are not shown because these were carried out on a more inclusive data set (Tomaszewska et al., in preparation)).

The five MSAs of nucleotides (CADMC: 693 bp, CADXM: 735 bp, TOPO: 678 bp, WGL: 420 bp, COI: 765 bp) were concatenated to form a supermatrix (five-fragment MSAs, Additional file 3: Supermatrix S1, 52 sequences, 3291 columns and 1571 informative sites) with a custom Perl script, that also generates character sets corresponding to the concatenated gene boundaries (available on request from AZ). To explore potential conflicting phylogenetic signal between the individual gene fragments in the concatenation, each one was excluded in turn from the MSAs and the resulting four-fragment MSAs were analysed using ML as implemented in RAxML (v8.0.26; [42]) (Additional file 4: Fig. S1a–e). The best ML topology and support values from 100 rapid bootstrap pseudo-replicates were compared to the analysis results of the five-fragment MSAs, not showing conflict among well-supported nodes (bootstrap values >85%) between topologies.

We inferred the optimal substitution models and partitioning scheme with PartitionFinder (version 1.1.1; [43]) using data blocks by gene fragment (CADMC, CADXM, TOPO, WGL, COI) and codon position as input, applying a greedy search approach with branch lengths linked across partitions and the Bayesian Information Criterion (BIC). The best partitioning scheme with corresponding substitution models (Additional file 5: Table S2) was then used to infer phylogenetic trees under the ML optimality criterion, as implemented in GARLI (version 2.01; [44]) (Additional file 6: Fig. S4). A total of 1080 heuristic tree searches were carried out on CSIRO compute cluster system, Pearcey (Dell PowerEdge M630), and the tree with the highest likelihood score selected. Bootstrap support values were obtained from 500 non-parametric bootstrap replicates with 10 heuristic tree search replicates each. Bootstrap values were mapped onto the ML tree using SumTrees (DendroPy version 3.12.2; [45]) and visualised with FigTree (version 1.4.2; https://github.com/rambaut/figtree, accessed May 8, 2015). The data was also analysed using a Bayesian method, as implemented in MrBayes (version 3.2.6; [46]) and the BEAGLE library (version 2.1.2; [47]). All model parameters, except branch lengths, remained unlinked, and two independent phylogenetic analyses were run with four chains each, sampling for 10 million generations every 1000th generation. The standard deviation of split frequencies was found to be <0.01, and convergence of the two runs was assessed using Tracer (version 1.6.0; [48]). The first 25% of the sampled trees were discarded as burn-in and the remaining sampled trees from the two runs were pooled. A 50% majority rule consensus tree with clade frequencies (posterior probabilities) was calculated with SumTrees and printed with FigTree (Additional file 7: Fig. S2).

To check for potentially detrimental influence of synonymous substitutions, the five-fragment MSA was fully degenerated with their respective genetic codes, using the software Degeneracy Coding (version 1.4; [49]). The resulting degenerated MSAs was analysed using RAxML with the same setting as for the four-fragment MSAs data set (which refers to the five gene fragment MSA less one gene fragment) (Additional file 8: Fig. S3).

### Ancestral character state reconstruction

**Fig. 3:**
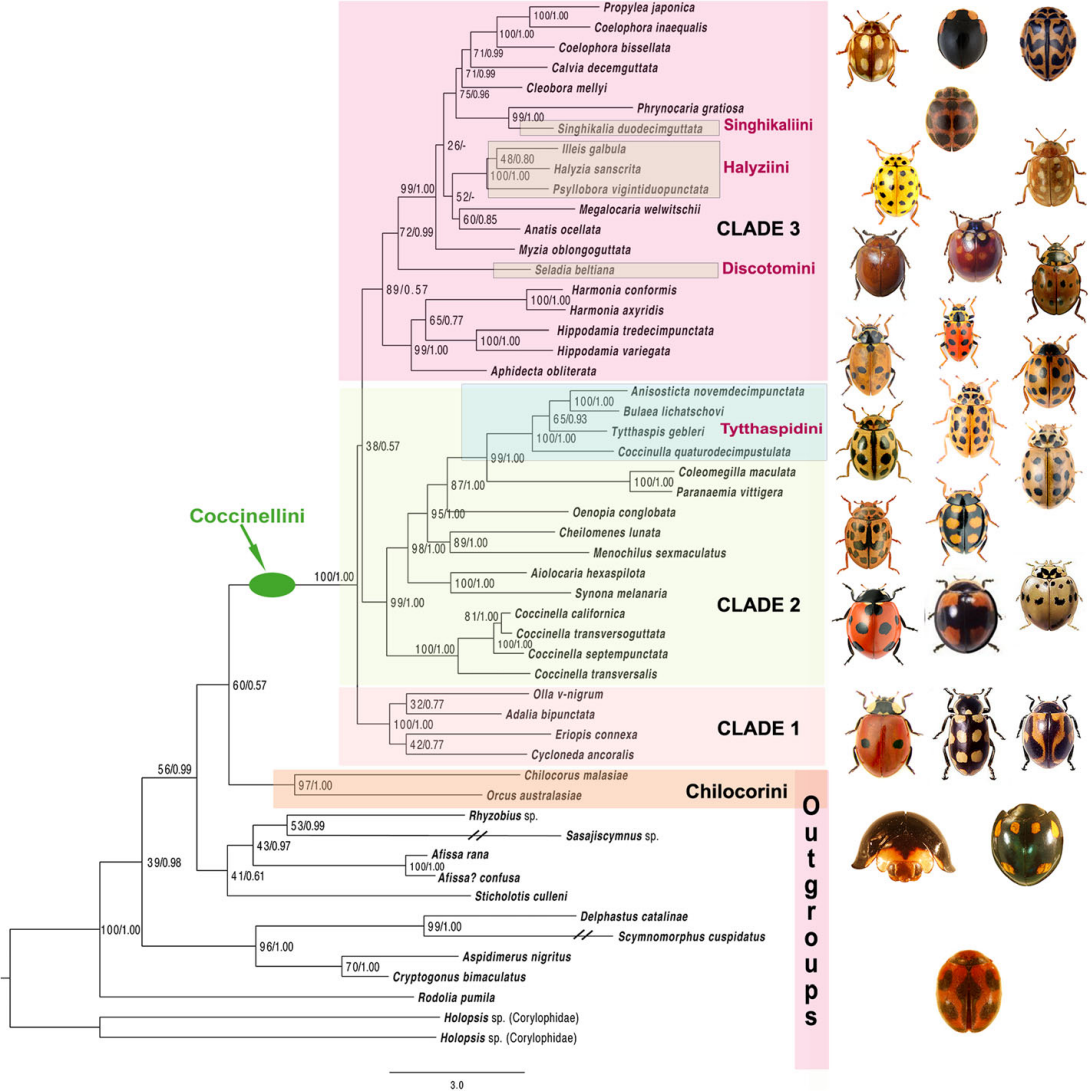
Phylogeny of Coccinellini based on ML best topology; number above branches show bootstrap support and posterior probability value above 0.50. Clades 1–3 of Coccinellini are discussed in the text. Taxa formerly classified in tribes Halyziini, Singhikaliini, Discotomini and Tytthaspidini are showed in different colour

The ancestral character states of six discrete morphological and of one behavioural character (Additional file 1: Table S3) were inferred using the maximum parsimony (MP) and ML methods as implemented in Mesquite (version 3.1; [50]) and using the ML tree (Fig. 3) as backbone. The Mk1 model, also implemented in Mesquite, was used to calculate the ML probabilities of the ancestral states.

## Results

### Phylogenetic analyses

The ML and Bayesian phylogenetic analyses of the five-fragment MSA resulted in identical topologies (Fig. 3, ML topology, log likelihood −55,518.302879 and Additional file 7: Fig. S2, the result from the Bayesian analysis). In both cases, the topology is mostly well supported, with 30 of 49 edges having a bootstrap support value of at least 75% and 35 of 49 edges having a posterior probability of at least 0.95 (both here subjectively regarded as “at least moderately supported”).

The ML analysis of the degeneracy-coded five-fragment MSA resulted in a similar topology with 21 of 49 edges at least moderately supported (Additional file 8: Fig. S3). These 21 edges were also all present in the above topology generated from the non-degenerated data (Fig. 3). Except for the sister-group relationship between Coccinellini and Chilocorini (bootstrap values of 76% and 60% with the degenerated and non-degenerated data, respectively), support values from the degenerated data are not much higher than those from analysing the non-degenerated data set. The higher bootstraps support values of the analysis with non-degenerated data and the topological congruence between results based on non-degenerated (Fig. 3) and degenerated data (Additional file 8: Fig. S3), for at least 21 edges with moderate to very strong support, are both indicative of the utility of the synonymous changes for the estimation of the Coccinellini phylogeny. The subsequent discussion, therefore, focuses on analyses of the non-degenerated data set (Fig. 3).

#### Coccinellini – Monophyly and sister relationship

To assess the support for the monophyly of Coccinellini, we used a comprehensive taxon sampling that represents all previously recognized tribes of Coccinellinae: Coccinellini, Discotomini, Halyziini, Singhikaliini, and Tytthaspidini (incl. Bulaeini). The outgroup includes twelve species of ladybirds classified as Microweiseinae (two species) and the Coccinellinae tribes Chilocorini (two species), Epilachnini (two species), Aspidimerini (two species), Noviini (one species), Sticholotidini (one species) and Coccidulini (two species). In addition, we included two species of fungivorous Corylophidae as more distant outgroup taxa within the superfamily Coccinelloidea [2] (Additional file 9: Table S4). The monophyly of Coccinellini sensu *lato* [13] was strongly supported with a bootstrap value of 100% and a posterior probability of 1.0.

Despite recent attempts to establish phylogenetic relationships within Coccinellidae, there is still no satisfactory resolution within the broadly defined subfamily Coccinellinae, that would lead to a stable tribal classification [17]. In previous studies, Chilocorini and Coccinellini were repeatedly recovered as monophyletic groups and as sister taxa of each other [2, 15, 17]. Our results from the ML and Bayesian analyses are consistent with these findings, but the support is weak (Bootstrap Support (BS) 60%, Posterior Probability (PP) 0.57; Fig. 3). Only moderate support was obtained when analysing the degenerated data set (BS 76%; Additional file 8: Fig. S3). Other previously-suggested phylogenetic positions of Coccinellini [14, 16] were not supported in our analyses.

#### Major clades within the tribe

Our analyses recovered three strongly supported clades within Coccinellini (Fig. 3), but relationships between these clades remain unresolved, as they are connected by short edges with low support values (i.e., BS < = 38 and PP < = 0.57). Clade 1 consists of species of the widespread genus *Adalia* and of the three New World genera *Olla*, *Cycloneda*, and *Eriopis* that are speciose in Central and South America [51]. Clades 2 and 3 comprise large radiations of primarily Old World species. Clade 2 is composed of species of the Holarctic genus *Coccinella*, of species of several genera formerly included in Tytthaspidini of species of the Holarctic genera *Coleomegilla* and *Paranaemia* (sometimes classified as Hippodamiini), and of species of the genera *Oenopia*, *Cheilomenes*, *Aiolocaria* and *Synona*. Within Clade 2, the genus *Coccinella* forms the sister group to the other species included in this clade. Clade 3 includes many genera. The Holarctic genera *Aphidecta* and *Hippodamia* and diverse Old World genus *Harmonia* constitute a well-supported group (BS 99%, PP 1.0). The Neotropical genus *Seladia* (formerly Discotomini) forms a moderately supported (BS 72%, PP 0.99) sister group to a phylogenetically unresolved complex of genera (BS 99%, PP 1.0) that includes the Old World *Cleobora*, *Coelophora*, *Propylea*, all genera of the former Halyziini, the Chinese species *Singhikalia duodecimguttata* (former Singhikaliini) (BS 99%, PP 1.0), and the widely-distributed Indo-Australian species *Phrynocaria gratiosa*. Interestingly, very large species of Coccinellini feeding on Hemiptera, *Anatis ocellata* (aphids) and *Megalocaria* (heteropterans), form a sister, albeit weakly supported (BS 52%, PP < = 0.50) group to powdery mildew fungi feeding taxa of the former Halyziini (*Halyzia, Illeis* and *Psyllobora*).

#### Ancestral state reconstruction

The results from the ancestral state reconstruction of adult pubescence, mandible type, female colleterial glands, larval dorsal gland openings, larval wax secretions, and pupal gin traps are presented on Additional files 10, 11, 12, 13, 14 and 15: Figs. S5–S10. Both the ML and MP approaches to ancestral state reconstruction are congruent and revealed that the female colleterial glands (Additional file 10: Fig. S5) and pupal gin traps (Additional file 11: Fig. S6) were most likely present in the most recent common ancestor of Coccinellini, strongly supporting the monophyletic origin of this clade. The the common ancestor of Chilocorini and Coccinellini lacked adult dorsal pubescence (Additional file 14: Fig. S9), but it was regained in *Singhikalia,* the only known genus of Coccinellini with dorsal pubescence. The highly agile larvae of Coccinellini lack both defensive gland openings and protective waxes (Additional files 12 and 13: Figs. S7 and S8), and the ancestral state reconstruction analyses indicate that these features were lost in the most recent common ancestor of Chilocorini and Coccinellini. In our data set, larval and pupal waxes are present in only a few genera of Coccidulini (*Rodolia*, *Sasajiscymnus*, *Rhyzobius*) and appear to have evolved convergently (Additional file 13: Fig. S8).

With respect to the food preferences and associated structural modifications of the adult mouth parts (Additional file 15: Fig. S10), the ancestral state reconstruction analyses suggest that preying upon aphids is the ancestral state of Coccinellini, and that feeding on other Hemiptera, beetle larvae, mildew, spores, pollen and plant tissue has occurred multiple times independently.

## Discussion

### Phylogenetic analyses

**Fig. 4:**
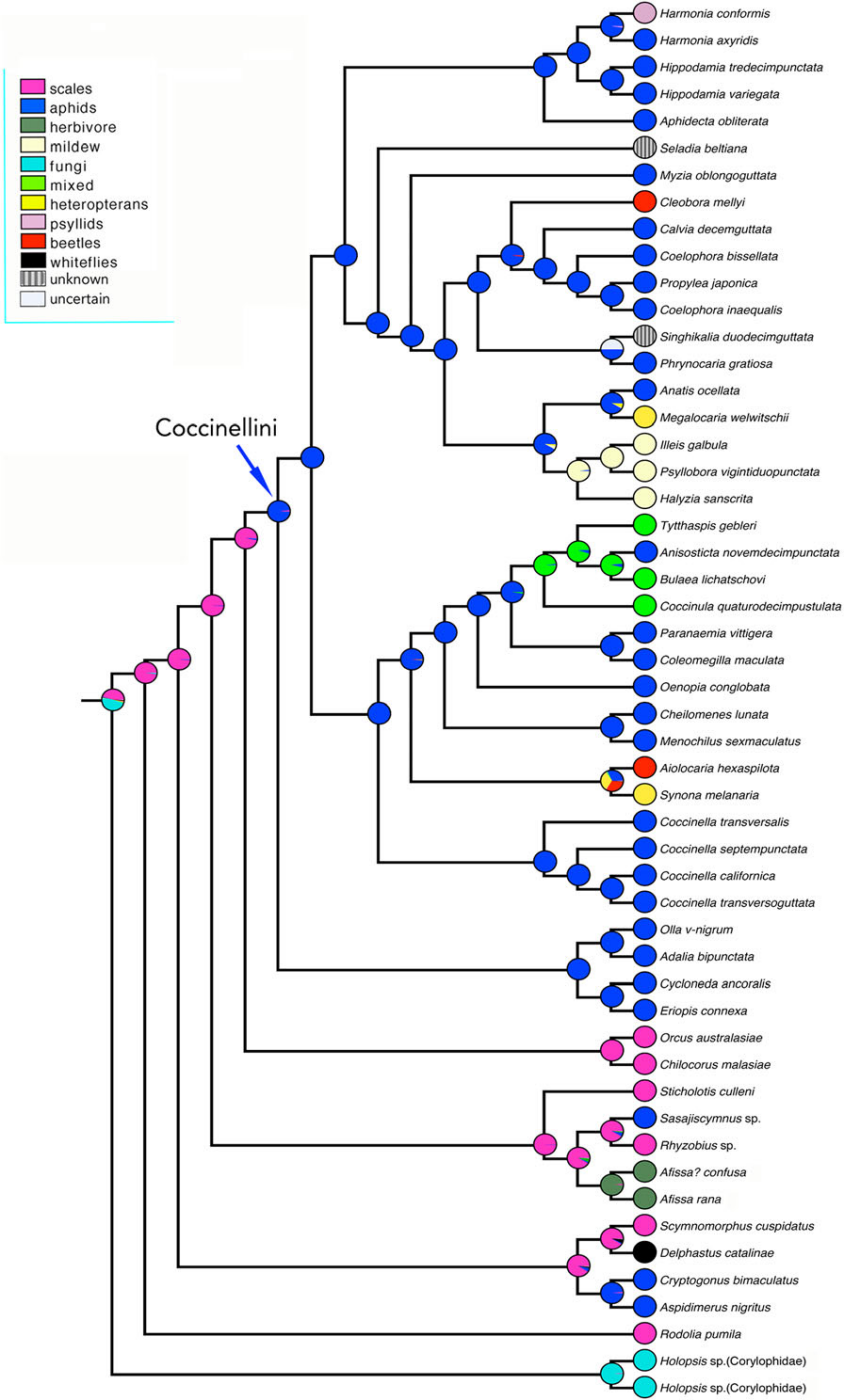
Ancestral state reconstruction of food preferences for the Coccinellini based on maximum likelihood method in Mesquite

In agreement with previously published molecular phylogenetic studies [2, 14–17] the monophyly of Coccinellini was resolved with high confidence in our analyses. Our studies are also consistent with the research based on nuclear and mitochondrial markers [2, 15, 17] recovering Chilocorini as the sister taxon of Coccinellini. The traditional idea of Coccinellini and Epilachnini being sister groups [9, 11], derived from studying morphological characters, remained unsupported by our analyses, as they were in other molecular analyses, which recovered Epilachnini at the base of the tree of Coccinellidae [15], within the taxa classified in Coccidulini (incl. Scymnini; [14, 16, 17]) or as sister to Coccidulini [52]. Our results (Fig. 4) suggest that the relatively large and aposematically coloured adults of aphid-feeding Coccinellini and herbivorous Epilachnini, both living on exposed surfaces and capable of strong reflex bleeding, are independently derived from smaller scale feeding ancestors. Epilachnini, which nest in our inferences within the “Coccidulinae” clade, retained densely pubescent bodies, while the last common ancestor of Coccinellini and Chilocorini lost this character (Additional file 14: Fig. S9), with the exception of the genus *Singhikalia* (former Singhikaliini), the only known pubescent Coccinellini. The genus *Singhikalia* is deeply nested within the tribe Coccinellini and represents an interesting case of convergence, possibly because it is mimicking local members of Epilachnini. *Singhikalia ornata* Kapur (India, Vietnam, Taiwan) and *S. duodecimguttata* Xiao (China) are reddish with black colour markings, while *S. latemarginata* (Bielawski) (Papua New Guinea) is almost entirely black. In this respect, all *Singhikalia* species match local members of Epilachnini very closely, to the extent that they are often found in the same series in museum collections, suggesting that they may co-occur in the same area and host plants.

The former tribe Halyziini forms strongly supported monophyletic group placed within the Clade 3 and comprises of speciose but very poorly defined Old World genera *Coelophora, Calvia, Phrynocaria*, and *Propylea*, the Asian *Singhikalia*, Old World *Megalocaria,* and species poor Holarctic genera *Myzia* and *Anatis*. In spite of taxon sampling differences the relationships between some of these taxa are in agreement with previous studies [2, 15, 16]. The second branch of the Clade 3 consists of Holarctic *Aphidecta* as a sister taxon to *Harmonia* and *Hippodamia*. The relationship between the last two genera has been recovered before [2, 16] but the placement of *Aphidecta* in this clade is a new position.

The exclusively Meso- and South American former tribe Discotomini is a poorly known group of 5 genera diagnosed by their strongly serrate or pectinate antennae. Their placement within traditional Coccinellinae has been uncertain and the molecular studies [2, 14] published so far placed Discotomini as a sister group to the remaining Coccinellini. The combined molecular and morphological analysis of Seago et al. [17] recovered *Seladia* as a sister group to the former Halyziini. We have recovered *Seladia* deeply embedded within Clade 3 at the base of large primarily Old World taxa, including former Halyziini.

The placement of the small Old World genera *Tytthaspis* and *Bulaea* in Coccinellini varied considerably in the past but their close affinity has been recognised by Iablokoff-Khnzorian [53], who pointed out similarities in male and female genitalia of several genera, later recognized as Tytthaspidini (= Bulaeini) by Kovář [9]. Most of the genera of former Tytthaspidini form strongly supported monophyletic group within the Clade 2 with *Oenopia* as the sister group, which is in agreement with Magro et al. [15].

The close relationships between *Olla, Adalia* and *Cycloneda* recovered in the Clade 1 has been suggested before [2, 15] but not the inclusion of the Neotropical *Eriopis* in this clade. Such arrangement suggests that the endemic New World genera and almost cosmopolitan *Adalia* have had long and independent evolutionary history from the much more diverse and speciose Coccinellini of the Old World.

As none of the previously recognised tribes, Discotomini, Halyziini, Singhikaliini, and Tytthaspidini, correspond with major clades within Coccinellini re-granting any of them subtribal status would render Coccinellini paraphyletic. Our results indicate that the newly-discovered clades, Clades 1–3 (Fig. 3), should receive recognition as formal taxonomic units, but it requires corroboration by analysis of a larger data set (Tomaszewska et al., in preparation).

### Ancestral state reconstruction

#### Morphological characters

Coccinellini are generally recognised by relatively large adults having glabrous and convex dorsal surfaces, often with aposematic colouration, rather long and feebly clavate antennae inserted in front of large eyes, and strongly expanded “securiform” terminal maxillary palpomeres and ‘handle and blade’ female coxal plates [9, 11]. Most adults of Coccinellini can be distinguished by a combination of the above listed characters, but these are known to occur in taxa classified in other coccinellid groups. Ślipiński [13] expanded the list of diagnostic characters of Coccinellini, arguing that the presence of large paired reservoirs, associated with female terminalia called “colleterial glands” (Fig. 2i) and the development of the “gin traps” between abdominal tergites of the pupa (Fig. 2j) were unique developments within Coccinellidae and may constitute autapomorphies of Coccinellini. The functions of both these structures are not well understood but the secretion of the colleterial glands have been linked to mating behaviour or egg deposition in batches [54] while the “gin traps” significantly contribute to the pupal defence [55] facilitating a quick body flicking and by creating sharp edges between segments to pinch legs or entire bodies of predatory and parasitic invertebrates to discourage oviposition or predation (Figs. 1c, 2e).

To test the hypotheses by Ślipiński [13], we traced the evolution of pupal gin traps and female colleterial glands along our main ML tree with Mesquite. Using MP and ML methods applied to character evolution, we found that the above-mentioned characters originated in the common ancestor of Coccinellini (Additional files 10 and 11: Figs. S5 and S6) and consequently regard these characters as autapomorphies of the tribe.

In addition to the above traits, we investigated the development of larval dorsal abdominal glands (Additional file 12: Fig. S7) and protective larval waxes (Additional file 13: Fig. S8), present in many groups of ladybirds, but absent in Coccinellini. The function and homology of the dorsal glands in larvae of Coccinelloidea has not been thoroughly investigated, but paired openings on abdominal tergites are present in larvae of most Corylophidae, some Endomychidae and Coccinellidae [13]. They are absent in several ladybird groups, including Coccinellini. Adults of Coccinellidae are known to reflex-bleed by excreting droplets of alkaloid loaded hemolymph to deter or tangle apparent predators. This process is less studied in ladybird larvae, but the “bleeding” from the dorsal glands has been observed in *Hyperaspis maindroni* Sicard (J. Poorani, ICAR-NRCB, India, personal information) and *Orcus bilunnulatus* (Chilocorini, A. Ślipiński, personal observation). Larvae of several species of Coccinellini have not been observed to excrete hemolymph when disturbed (A. Ślipiński, personal observation). However, this process has been documented in larvae of *Harmonia axyridis* [56, 57], with droplets originating from intersegmental membranes on most abdominal segments, and more recently in larvae of *Hippodamia variegata* (O. Nedvěd, personal observation). It is unclear whether this is a species-specific behaviour or whether it has been overlooked in other Coccinellini.

The generalised carnivorous type of the adult mandible with bifid apex and molar part bearing two unequal teeth [9], that has originated in the ancestor of Coccinellidae has been carried over with very little modification in all predatory lineages of ladybirds with several independent origins of a single sharp apical incisor in specialized scale predators (Chilocorini, Microweiseinae). All known Coccinellini have apically bifid mandibles, used to pierce their prey, suck body fluids or to masticate the entire prey [26]. The mildew or microphagous feeding taxa (former Halyziini and Tytthaspidini) have the same type of mandible with additional serration along the incisor edge (Halyziini) or relatively stiff and comb like prostheca used to scoop the spores and pollen (Tytthaspidini). Interestingly, the mandible of sometimes phytophagous *Bulaea* does not differ from the microphagous type found in *Tytthaspis*, but markedly differs from strongly modified mandibles in phytophagous Epilachnini.

#### Food preferences

The evolution of food preferences in Coccinellidae is a very complex issue that has received much attention due to the importance of ladybirds as biological control agents [14, 58] and, more recently, due to the recognition of the environmental impact of introduced or invading ladybirds [59] on populations of native species. Some groups of ladybirds (e.g., Noviini, Stethorini, Telsimiini and most Chilocorini) show remarkably stable food preferences, feeding mostly on taxonomically narrow groups of invertebrates [22]. Coccidophagy, preying upon scale insects, which are gregarious organisms of limited mobility, has been evolved as an ancestral food preference in Coccinellidae [14, 17]. Coccidophagous coccinellids are often morphologically and physiologically adapted to a given prey [27, 60]. But even very specialized ladybirds feed and develop occasionally on a very different host (e.g., Stethorini, which are specialised on spider mites (Tetranychidae) can develop on whiteflies [61]).

Most species of Coccinellini are “general predators”, feeding in principle on aphids. Character-state reconstruction indicates that the transition from feeding on coccids to aphidophagy was acquired in the ancestor of Coccinellini (Fig. 4), but this feeding preference has independently arisen a few times in Coccinellinae (e.g., in Aspidimerini), in some genera of Coccidulini (e.g., *Coccidula*, *Sasajiscymnus*), Scymnini (*Scymnus*), and Platynaspidini (*Platynaspis*).

Coccinellini have diverse food preferences. While being primarily aphidophagous, they consume a broad spectrum of food that also includes other invertebrates, pollen, nectar, and often spores [14]. These opportunistic predators regularly cannibalize eggs and larvae of ladybirds, including those of their own species, and change diet depending on season and availability of prey. The gut contents of many species of Coccinellini examined during this study often consisted of predominantly sternorrhynchan Hemiptera mixed with pollen, and sometimes, with fungal spores.

Within Coccinellini, our results revealed repeated and phylogenetically independent food preference transitions from aphidophagy to other food sources (Fig. 4 and Additional file 16: Fig. S11): (a) to specialized and obligate mycophagy in the taxa classified in the former tribes Halyziini (*Halyzia*, *Illeis, Psyllobora*), feeding on hymenium and conidia of powdery mildew fungi (Erysiphales); (b) to a mixed diet in *Bulaea, Coccinula* and *Tytthaspis* (Tytthaspidini), with their known diet including spores of various Ascomycete fungi [32, 62], but also plant tissue (*Bulaea*), pollen (mainly of Asteraceae in *Coccinula*), acari and Thysanoptera (*Tytthaspis*); (c) to specialised predation on nymphs of the plataspid bugs in various phylogenetically independent lineages of some *Megalocaria* [63] and of *Synona* [64]; (d) to predation on larvae of Chrysomelidae in at least Asian *Aiolocaria* [65], Australian *Cleobora mellyi* [66] and the New World *Neoharmonia* sp. [67] (N. Vandenberg, USDA-Smithsonian, USA, personal communication; not included in our data), and (e) to psyllids (Psylloidea) as the essential food of *Harmonia conformis* at least in some geographic areas [68].

## Conclusions

This study represents the first molecular phylogenetic analysis of the tribe Coccinellini with a world-wide taxonomic sampling. Our phylogenetic analyses revealed strong support for Coccinellini sensu *lato* [13] being monophyletic and a sister group to Chilocorini. Three major clades were identified within Coccinellini, suggesting that Old and New World taxa, especially South American Coccinellini, have probably evolved separately. None of the major clades correspond to the previously recognised tribes Discotomini, Halyziini, Singhikaliini, or Tytthaspidini. Consequently, we suggest that these taxonomic units should no longer be used. Further testing with more taxa, especially from South America, is required to corroborate the constitution of and relationships between the three major clades of Coccinellini proposed in this study. Our study also provides an understanding of the diversification of Coccinellini and character evolution within this tribe, particularly the evolution of food preferences. The switch from obligate coccidophagy to aphidophagy in ancestral Coccinellini was accompanied by larval changes (losing dorsal defensive glands and strong dorsal ornamentation) for increased agility, and the pupae shedding larval skins completely and exposing dorsal gin traps.

## Additional files

Additional file 1: Table S3. Character states of six discrete morphological and of one behavioural character. (DOCX 29 kb)

Additional file 2: Table S1. Primers used for PCR amplification of the genes. (DOCX 17 kb)

Additional file 3: Supermatrix S1. The nucleotide multiple sequence alignment (MSA), used for the phylogenetic analysis, its partitions, nucleotide composition information and percentage of gaps and ambiguities per taxa. It contains 52 taxa identified by the species name, their voucher number and the gene fragments: WGL, TOPO, COI, CADXM and CADMC. (NEX 171 kb)

Additional file 4: Figs. S1a–e. The best RAxML ML trees with bootstrap values from 100 rapid bootstrap pseudo-replicates for the four fragment MSA that lack WGL (Fig. S1a), TOPO (Fig. S1b), COI (Fig. S1c), CADXM (Fig. S1d) and CADMC (Fig. S1e). (PDF 5145 kb)

Additional file 5: Table S2. The best (BIC) multiple sequence alignment partitioning scheme with corresponding substitution models generated with PartitionFinder (version 1.1.1; [41]). (TXT 2 kb)

Additional file 6: Fig. S4. The best GARLI ML tree for the non-degenerated five-fragment MSA set with bootstrap support values (equivalent to Fig. 3 without Posterior Probabilities). (PDF 127 kb)

Additional file 7: Fig. S2. The resulting tree of the MrBayes Bayesian analysis with posterior probabilities. (PDF 132 kb)

Additional file 8: Fig. S3. The best RAxML ML tree with bootstrap values for the fully degenerated five-fragment MSA. (PDF 131 kb)

Additional file 9: Table S4. Specimens used in this study with voucher identification (Australian National Insect Collection), data, and corresponding GenBank accession numbers. (XLS 35 kb)

Additional file 10: Fig. S5. Ancestral state reconstruction based on parsimony (A) and maximum likelihood (B) for female colleterial glands in Coccinellidae. The ancestral states are present (blue) and absent (green). The topology is derived from the ML tree in Fig. 3. (TIFF 39987 kb)

Additional file 11: Fig. S6. Ancestral state reconstruction based on parsimony (A) and maximum likelihood (B) for pupal gin traps in Coccinellidae. The ancestral states are present (green), absent (blue), unknown (shadowed) and uncertain (grey). The topology is derived from the ML tree in Fig. 3. (TIFF 53439 kb)

Additional file 12: Fig. S7. Ancestral state reconstruction based on parsimony (A) and maximum likelihood (B) for larval dorsal glands in Coccinellidae. The ancestral states are present (blue), absent (green), unknown (shadowed) and uncertain (grey). The topology is derived from the ML tree in Fig. 3. (TIFF 60213 kb)

Additional file 13: Fig. S8. Ancestral state reconstruction based on parsimony (A) and maximum likelihood (B) for larval waxes in Coccinellidae. The ancestral states are present (blue), absent (green), unknown (shadowed) and uncertain (grey). The topology is derived from the ML tree in Fig. 3. (TIFF 41014 kb)

Additional file 14: Fig. S9. Ancestral state reconstruction based on parsimony (A) and maximum likelihood (B) for presence of dorsal pubescence in Coccinellidae. The ancestral states are present (blue) and absent (green). The topology is derived from the ML tree in Fig. 3. (TIFF 59853 kb)

Additional file 15: Fig. S10. Ancestral state reconstruction based on parsimony (A) and maximum likelihood (B) for mandible type in Coccinellidae. The ancestral states are separated on fungivorous (light blue), mildew (purple), carnivorous1 (red), microphagous (yellow), phytophagous (green), carnivorous2 (black). The topology is derived from the ML tree in Fig. 3. (TIFF 60492 kb)

Additional file 16: Fig. S11. Ancestral state reconstruction based on parsimony (A) and maximum likelihood (B) for food preferences in Coccinellidae. The ancestral states are separated on scales (purple), aphids (navy blue), herbivore (blue), mildew (light green), fungi (dark green), mixed (light blue), heteropterans (yellow), psyllids (orange), beetles (red), whiteflies (black), unknown (shadowed), uncertain (grey). The topology is derived from the ML tree in Fig. 3. (TIFF 58415 kb)
