# Supplementary material for: Molecular phylogeny reveals food plasticity in the evolution of true ladybird beetles (Coleoptera: Coccinellidae: Coccinellini)
Source: BMC Evol Biol. 2017 Jun 26;17:151. doi: 10.1186/s12862-017-1002-3 (PMC5485688; doi:10.1186/s12862-017-1002-3)
Supplement: Supplementary file 1 — Character states of six discrete morphological and of one behavioural character. (DOCX 29 kb) [file 12862_2017_1002_MOESM1_ESM.docx]

| TAXON | Food preference | Female glands | Larval glands | Pupa gin traps | Adult pubescence | Larval waxes | Mandible type | Food preference | Female glands | Larval glands | Pupa gin trap | Adult pubescence | Larval waxes | Mandible type |
| --- | --- | --- | --- | --- | --- | --- | --- | --- | --- | --- | --- | --- | --- | --- |
| *Aiolocaria hexaspilota* | beetles | yes | no | yes | no | no | carnivorous1 | 8 | 0 | 1 | 1 | 1 | 1 | 2 |
| *Synona melanaria* | heteropterans | yes | no | yes | no | no | carnivorous1 | 6 | 0 | 1 | 1 | 1 | 1 | 2 |
| *Oenopia conglobata* | aphids | yes | no | yes | no | no | carnivorous1 | 1 | 0 | 1 | 1 | 1 | 1 | 2 |
| *Chilocorus malasiae* | scales | no | yes | no | no | no | carnivorous2 | 0 | 1 | 0 | 0 | 1 | 1 | 5 |
| *Cheilomenes lunata* | aphids | yes | no | yes | no | no | carnivorous1 | 1 | 0 | 1 | 1 | 1 | 1 | 2 |
| *Menochilus sexmaculatus* | aphids | yes | no | yes | no | no | carnivorous1 | 1 | 0 | 1 | 1 | 1 | 1 | 2 |
| *Tytthaspis gebleri* | mixed | yes | no | yes | no | no | microphagous | 5 | 0 | 1 | 1 | 1 | 1 | 3 |
| *Bulaea lichatschovi* | mixed | yes | no | yes | no | no | microphagous | 5 | 0 | 1 | 1 | 1 | 1 | 3 |
| *Scymnomorphus cuspidatus* | scales | no | no | no | yes | no | carnivorous2 | 0 | 1 | 1 | 0 | 0 | 1 | 5 |
| *Anisosticta novemdecimpunctata* | aphids | yes | no | yes | no | no | carnivorous1 | 1 | 0 | 1 | 1 | 1 | 1 | 2 |
| *Coleomegilla maculata* | aphids | yes | no | yes | no | no | carnivorous1 | 1 | 0 | 1 | 1 | 1 | 1 | 2 |
| *Calvia decemguttata* | aphids | yes | no | yes | no | no | carnivorous1 | 1 | 0 | 1 | 1 | 1 | 1 | 2 |
| *Coccinella transversalis* | aphids | yes | no | yes | no | no | carnivorous1 | 1 | 0 | 1 | 1 | 1 | 1 | 2 |
| *Harmonia conformis* | psyllids | yes | no | yes | no | no | carnivorous1 | 7 | 0 | 1 | 1 | 1 | 1 | 2 |
| *Hippodamia variegata* | aphids | yes | no** | yes | no | no | carnivorous1 | 1 | 0 | 1 | 1 | 1 | 1 | 2 |
| *Holopsis* sp. ex Tasmania (Corylophidae) | fungi | no | yes | no | yes | no | fungivorous | 4 | 1 | 0 | 0 | 0 | 1 | 0 |
| *Holopsis* sp. ex AU, ACT (Corylophidae) | fungi | no | yes | no | yes | no | fungivorous | 4 | 1 | 0 | 0 | 0 | 1 | 0 |
| *Psyllobora vigintiduopunctata* | mildew | yes | no | yes | no | no | mildew | 3 | 0 | 1 | 1 | 1 | 1 | 1 |
| *Aphidecta obliterata* | aphids | yes | no | yes | no | no | carnivorous1 | 1 | 0 | 1 | 1 | 1 | 1 | 2 |
| *Aspidimerus nigritus* | aphids | no | yes | no | yes | no | carnivorous1 | 1 | 1 | 0 | 0 | 0 | 1 | 2 |
| *Cryptogonus bimaculatus* | aphids | no | yes | no | yes | no | carnivorous1 | 1 | 1 | 0 | 0 | 0 | 1 | 2 |
| *Sticholotis culleni* | scales | no | yes | no | no | no | carnivorous1 | 0 | 1 | 0 | 0 | 1 | 1 | 2 |
| *Sasajiscymnus* sp. ex China | aphids | no | no | no | yes | yes | carnivorous1 | 1 | 1 | 1 | 0 | 0 | 0 | 2 |
| *Rhyzobius* sp. ex AU | scales | no | no | no | yes | yes | carnivorous1 | 0 | 1 | 1 | 0 | 0 | 0 | 2 |
| *Rodolia pumila* | scales | no | no | no | yes | yes | carnivorous1 | 0 | 1 | 1 | 0 | 0 | 0 | 2 |
| *Singhikalia duodecimguttata* | ? | yes | ? | ? | yes | ? | carnivorous1 | ? | 0 | ? | ? | 0 | ? | 2 |
| *Coccinella septempunctata* | aphids | yes | no | yes | no | no | carnivorous1 | 1 | 0 | 1 | 1 | 1 | 1 | 2 |
| *Coccinella transversoguttata* | aphids | yes | no | yes | no | no | carnivorous1 | 1 | 0 | 1 | 1 | 1 | 1 | 2 |
| *Adalia bipunctata* ex USA | aphids | yes | no | yes | no | no | carnivorous1 | 1 | 0 | 1 | 1 | 1 | 1 | 2 |
| *Eriopis connexa* | aphids | yes | no | yes | no | no | carnivorous1 | 1 | 0 | 1 | 1 | 1 | 1 | 2 |
| *Orcus australasiae* | scales | no | yes | no | no | no | phytophagous | 0 | 1 | 0 | 0 | 1 | 1 | 4 |
| *Harmonia axyridis* | aphids | yes | no** | yes | no | no | carnivorous1 | 1 | 0 | 1 | 1 | 1 | 1 | 2 |
| *Hippodamia tredecimpunctata* | aphids | yes | no | yes | no | no | carnivorous1 | 1 | 0 | 1 | 1 | 1 | 1 | 2 |
| *Myzia oblongoguttata* | aphids* | yes | no | yes | no | no | carnivorous1 | 1 | 0 | 1 | 1 | 1 | 1 | 2 |
| *Afissa rana* | herbivore | no | no | no | yes | no | phytophagous | 2 | 1 | 1 | 0 | 0 | 1 | 4 |
| *Halyzia sanscrita* | mildew | yes | no | yes | no | no | mildew | 3 | 0 | 1 | 1 | 1 | 1 | 1 |
| *Megalocaria welwitschii* | heteropterans | yes | no | yes | no | no | carnivorous1 | 6 | 0 | 1 | 1 | 1 | 1 | 2 |
| *Afissa? confusa* | herbivore | no | no | no | yes | no | phytophagous | 2 | 1 | 1 | 0 | 0 | 1 | 4 |
| *Phrynocaria gratiosa* | aphids | yes | no | yes | no | no | carnivorous1 | 1 | 0 | 1 | 1 | 1 | 1 | 2 |
| *Coelophora bissellata* | aphids | yes | no | yes | no | no | carnivorous1 | 1 | 0 | 1 | 1 | 1 | 1 | 2 |
| *Propylea japonica* | aphids | yes | no | yes | no | no | carnivorous1 | 1 | 0 | 1 | 1 | 1 | 1 | 2 |
| *Cleobora mellyi* | beetles | yes | no | yes | no | no | carnivorous1 | 8 | 0 | 1 | 1 | 1 | 1 | 2 |
| *Delphastus catalinae* | whiteflies | no | no | no | no | no | carnivorous2 | 9 | 1 | 1 | 0 | 1 | 1 | 5 |
| *Coelophora inaequalis* | aphids | yes | no | yes | no | no | carnivorous1 | 1 | 0 | 1 | 1 | 1 | 1 | 2 |
| *Cycloneda ancoralis* | aphids | yes | no | yes | no | no | carnivorous1 | 1 | 0 | 1 | 1 | 1 | 1 | 2 |
| *Coccinella californica* | aphids | yes | no | yes | no | no | carnivorous1 | 1 | 0 | 1 | 1 | 1 | 1 | 2 |
| *Olla v-nigrum* | aphids | yes | no | yes | no | no | carnivorous1 | 1 | 0 | 1 | 1 | 1 | 1 | 2 |
| *Paranaemia vittigera* | aphids | yes | no | yes | no | no | carnivorous1 | 1 | 0 | 1 | 1 | 1 | 1 | 2 |
| *Illeis galbula* ex PNG | mildew | yes | no | yes | no | no | mildew | 3 | 0 | 1 | 1 | 1 | 1 | 1 |
| *Coccinula quatuordecimpustulata* | aphids | yes | no | yes | no | no | carnivorous1 | 1 | 0 | 1 | 1 | 1 | 1 | 2 |
| *Anatis ocellata* | aphids | yes | no | yes | no | no | carnivorous1 | 1 | 0 | 1 | 1 | 1 | 1 | 2 |
| *Seladia beltiana* | ? | yes | ? | ? | no | ? | carnivorous1 | ? | 0 | ? | ? | 1 | ? | 2 |

** droplets have been reported in the abdomen of larvae of *Harmonia axyridis* [51, 52] and *Hippodamia variegata* (Oldřich Nedvěd pers. comment) but no correlation with a gland have been found yet.
