# Supplementary material for: Molecular phylogeny reveals food plasticity in the evolution of true ladybird beetles (Coleoptera: Coccinellidae: Coccinellini)
Source: BMC Evol Biol. 2017 Jun 26;17:151. doi: 10.1186/s12862-017-1002-3 (PMC5485688; doi:10.1186/s12862-017-1002-3)
Supplement: Supplementary file 2 — Primers used for PCR amplification of the genes. (DOCX 17 kb) [file 12862_2017_1002_MOESM2_ESM.docx]

| **Primer Name** | **M13 tail in italics + primer sequence 5’→3’** | **Reference** |
| --- | --- | --- |
| **M13** |  |  |
| M13REV | *CAGGAAACAGCTATGACC* | [65] |
| M13(-21) | *TGTAAAACGACGGCCAGT* | [65] |
|  |  |  |
| **CO1** |  |  |
| Jerry_t | *CAGGAAACAGCTATGACC* CAACATTTATTTTGATTTTTTGG | [64] |
| Superpat_t | *TGTAAAACGACGGCCAGT* GCACATWTCTGCCATATTAGA |  |
|  |  |  |
| **CAD** |  |  |
| CAD-MC  Round 1 |  |  |
| CD439F_t | *CAGGAAACAGCTATGACC* TTCAGTGTACARTTYCAYCCHGARCAYAC | [41] |
| CD688R_t | *TGTAAAACGACGGCCAGT* TGTATACCTAGAGGATCDACRTTYTCCATRTTRCA | [41] |
| CAD-MC  Round 2 |  |  |
| CD439F_t | As Round 1 |  |
| CD668R_t | *TGTAAAACGACGGCCAGT* ACGACTTCATAYTCNACYTCYTTCCA | [41] |
|  |  |  |
| CAD-XM Round 1 |  |  |
| CD806F3_t | *CAGGAAACAGCTATGACC* TTAYTGYGTTGTNAARATWCCNMGNTGGGA | [41] |
| CD1098R2_t | *TGTAAAACGACGGCCAGT* GCTATGTTGTTNGGNAGYTGDCCNCCCAT | [41] |
| CAD-XM Round 2 |  |  |
| CD821F_t | *CAGGAAACAGCTATGACC* AGCACGAAAATHGGNAGYTCNATGAARAG | [41] |
| CD1098R2_t | As Round 1 |  |
|  |  |  |
| CAD-XM2 Round 1 |  |  |
| CD439F_t | *CAGGAAACAGCTATGACC* TTCAGTGTACARTTYCAYCCHGARCAYAC | [41] |
| CD1098R2_t | *TGTAAAACGACGGCCAGT* GCTATGTTGTTNGGNAGYTGDCCNCCCAT | [41] |
| CAD-XM2 Round 2 |  |  |
| CD93F | AGGAATACAC ACAGGAGAATC |  |
| CD1079R | CATCCGACAG CACACCAAT |  |
| **TOPO**  Round 1 |  |  |
| TP643F_t | *CAGGAAACAGCTATGACC* GACGATTGGAARTCNAARGARATG | [41] |
| TP932R_t | *TGTAAAACGACGGCCAGT* GGWCCDGCATCDATDGCCCA | [41] |
| TOPO  Round 2 |  |  |
| TP675F_t | *CAGGAAACAGCTATGACC* GAGGACCAAGCNGAYACNGTDGGTTGTTG | [41] |
| TP919R_t | *TGTAAAACGACGGCCAGT* GTCTCTTTGCGTYTTRTTRTADATYTTYTC | [41] |
|  |  |  |
| **Wingless** Round 1 |  |  |
| Wg550F_t | *CAGGAAACAGCTATGACC* ATGCGTCAGGARTGYAARTGYCAYGGYATGTC | [41] |
| WgAbRZ_t | *TGTAAAACGACGGCCAGT* CACTTNACYTCRCARCACCARTG | [41] |
| Wingless  Round 2 |  |  |
| Wg578F_t | *CAGGAAACAGCTATGACC* TGCACNGTGAARACYTGCTGGATG | [41] |
| WgAbR_t | *TGTAAAACGACGGCCAGT* ACYTCGCAGCACCARTGGAA | [41] |
|  |  |  |
| **3059fin** |  |  |
| 3059fin1F | *CAGGAAACAGCTATGACC* GGNATHCAYGARGARATG | [65] |
| 3059fin3R | *TGTAAAACGACGGCCAGT* TARAANACNGTYTGYTTCCARTG | [65] |

**Additional file 1: Table S1**
