# Supplementary figures and images for: Molecular phylogeny reveals food plasticity in the evolution of true ladybird beetles (Coleoptera: Coccinellidae: Coccinellini)
Source: BMC Evol Biol. 2017 Jun 26;17:151. doi: 10.1186/s12862-017-1002-3 (PMC5485688; doi:10.1186/s12862-017-1002-3)

Figure S1a

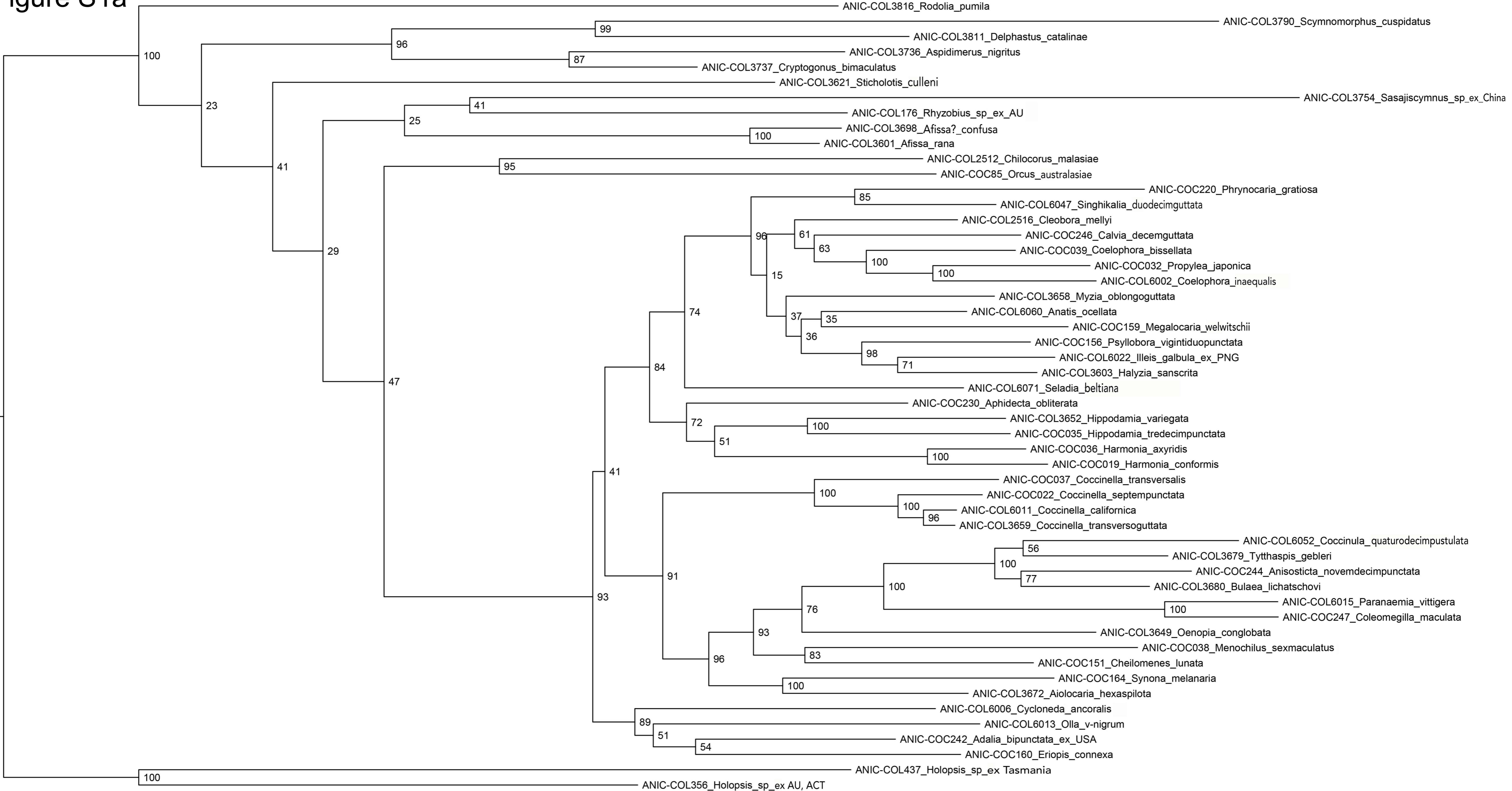

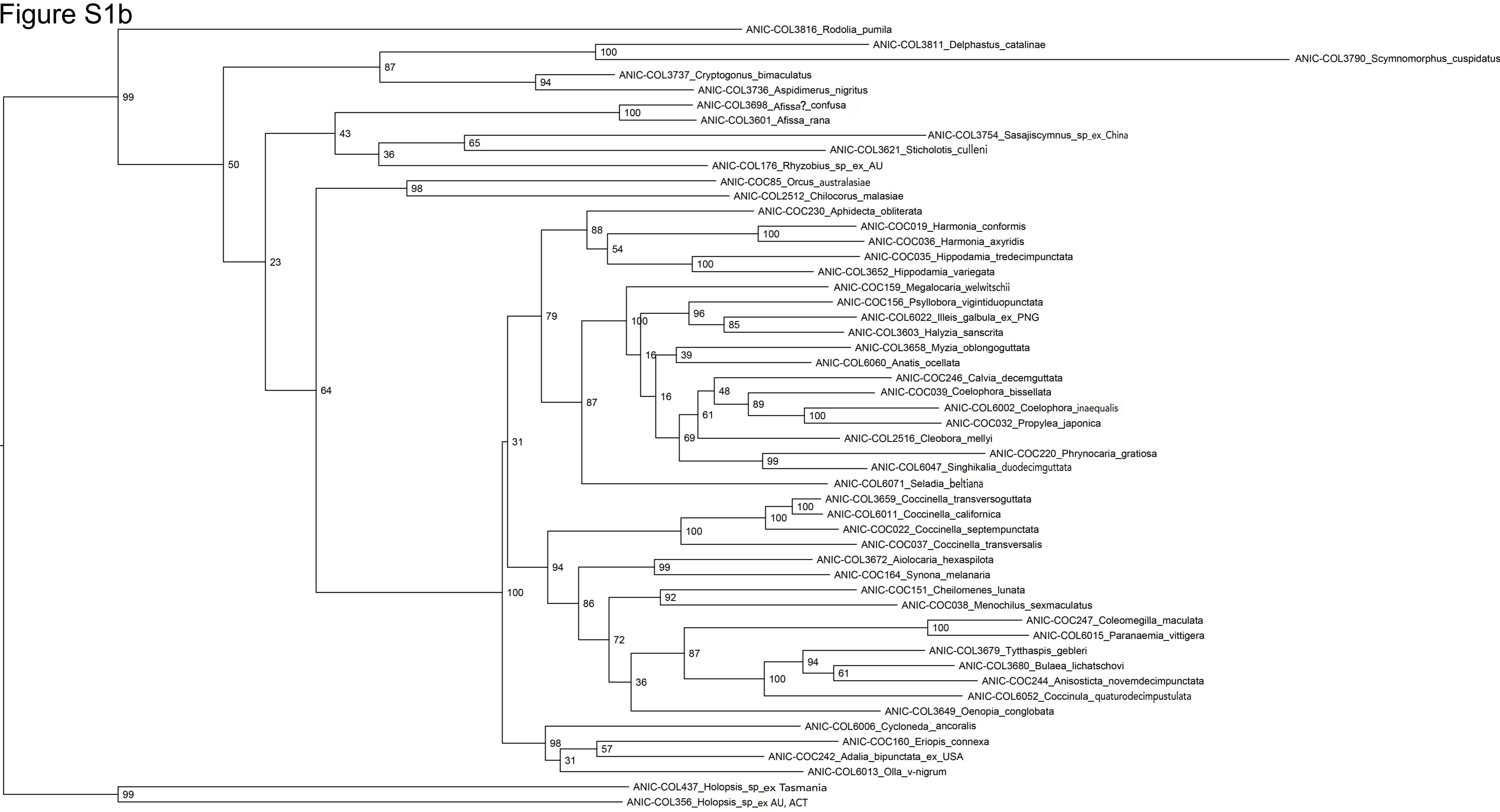

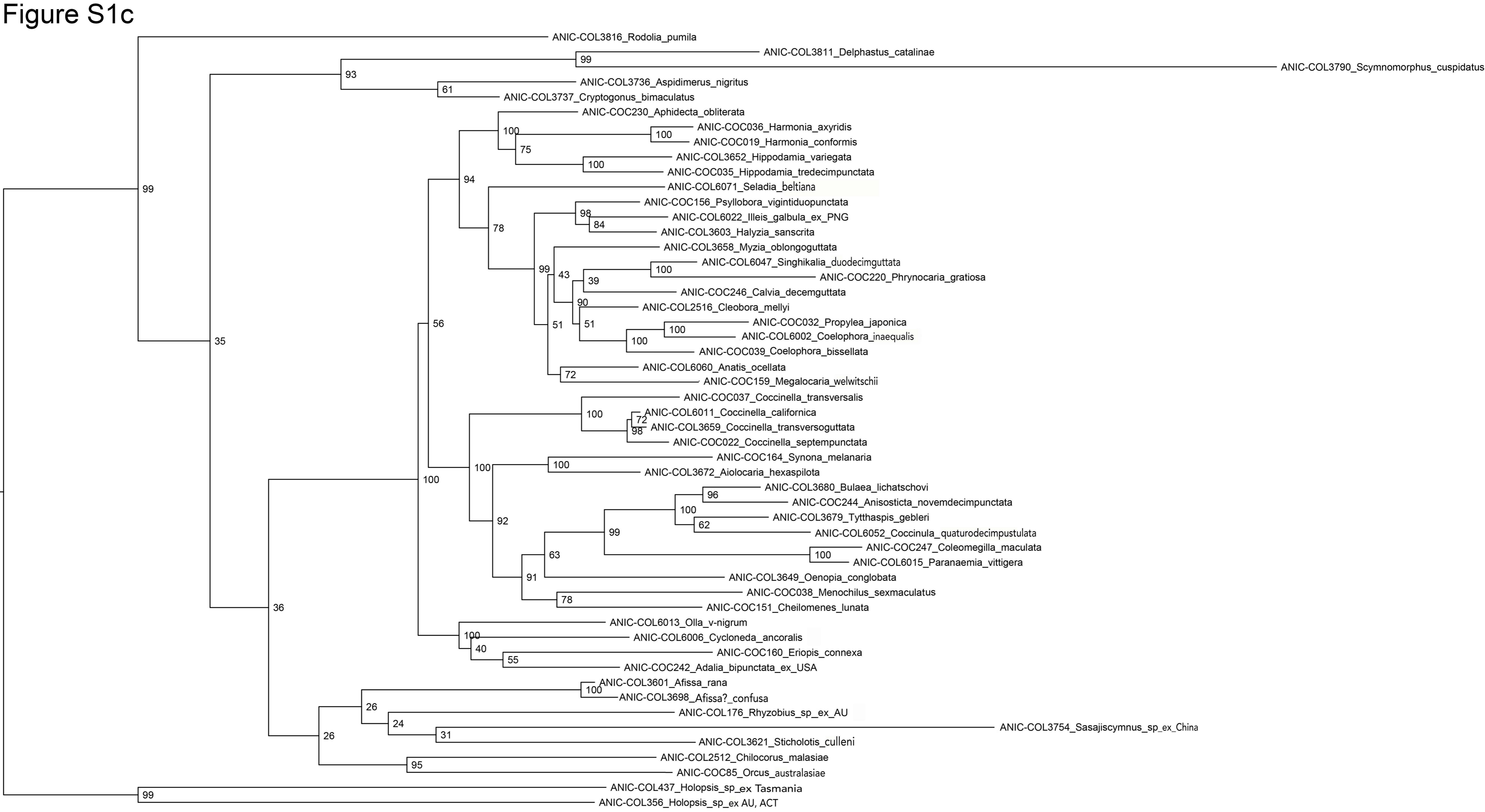

Figure S1d

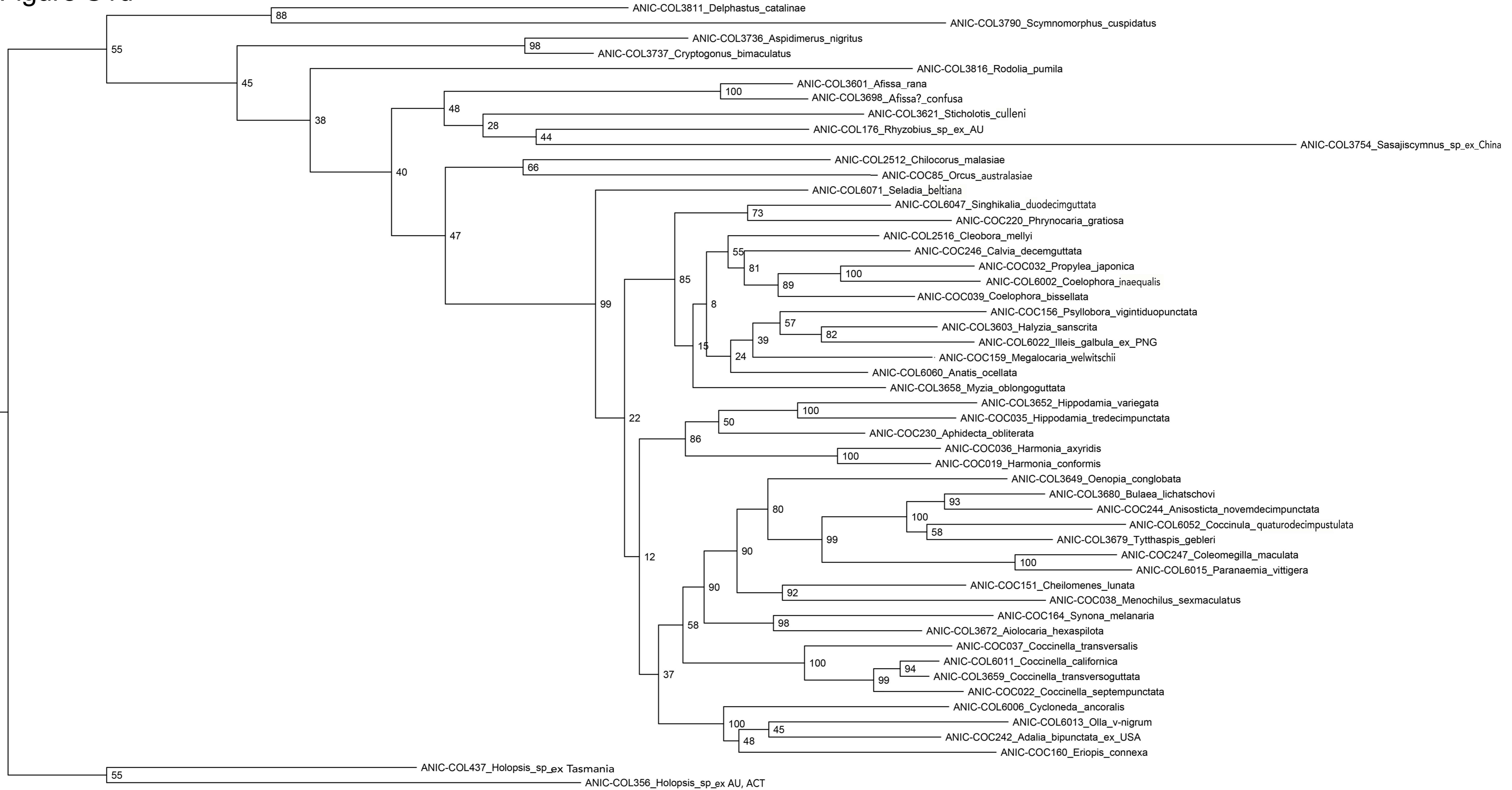

Figure S1e

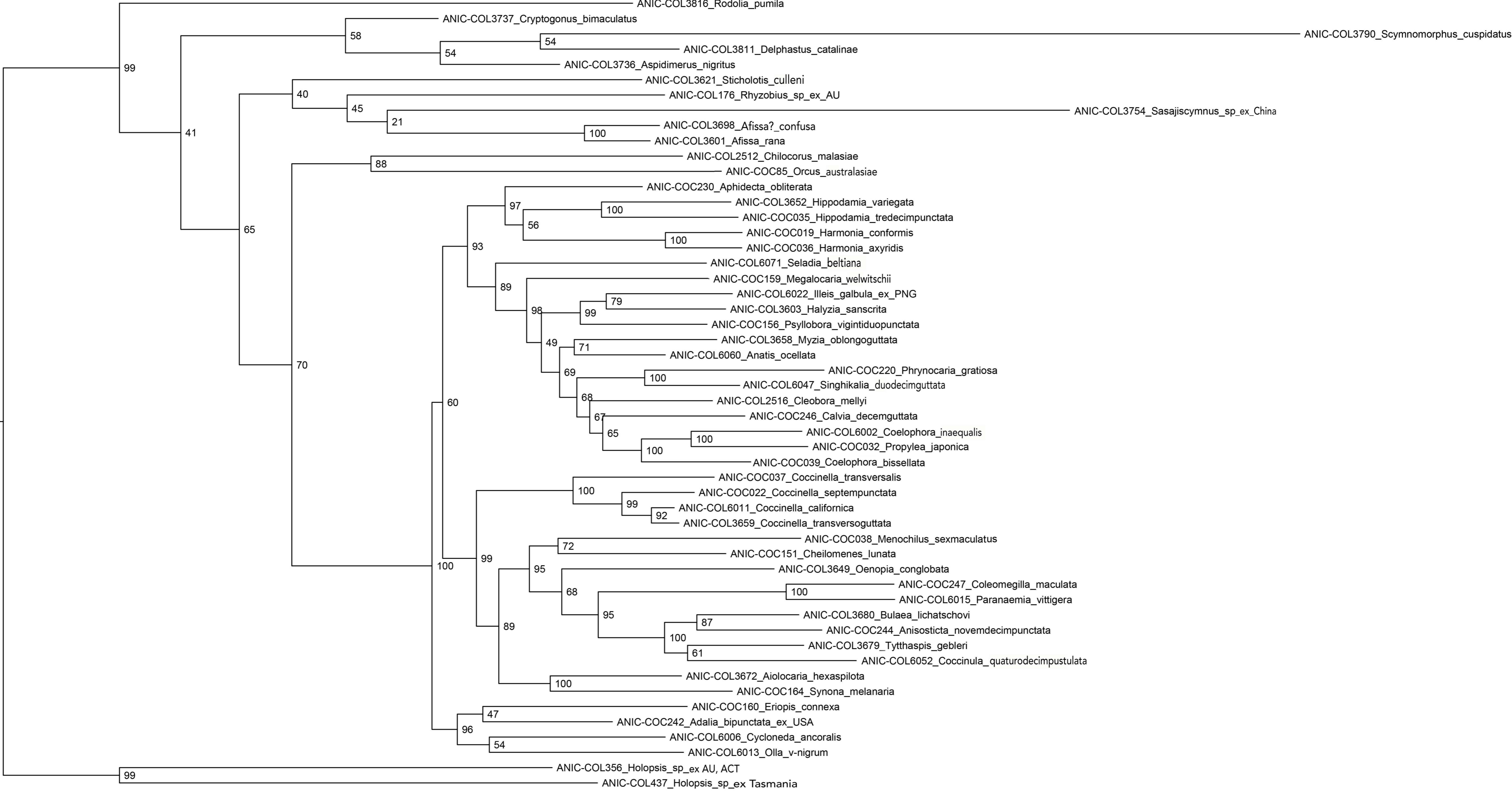

Supplement: Supplementary file 4 — The best RAxML ML trees with bootstrap values from 100 rapid bootstrap pseudo-replicates for the four fragment MSA that lack WGL (Fig. S1a), TOPO (Fig. S1b), COI (Fig. S1c), CADXM (Fig. S1d) and CADMC (Fig. S1e). (PDF 5145 kb) [file 12862_2017_1002_MOESM4_ESM.pdf]

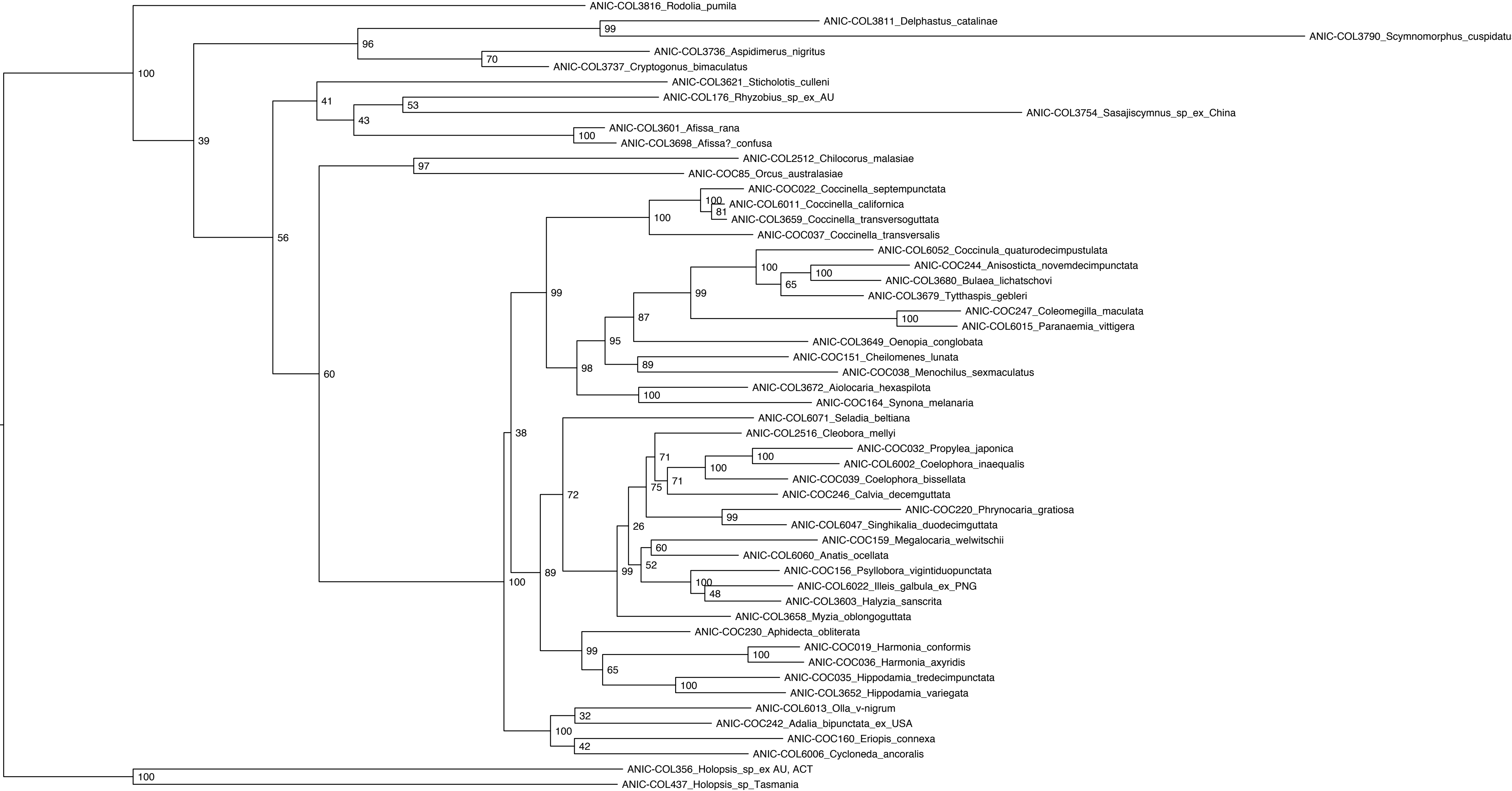

Supplement: Supplementary file 6 — The best GARLI ML tree for the non-degenerated five-fragment MSA set with bootstrap support values (equivalent to Fig. 3 without Posterior Probabilities). (PDF 127 kb) [file 12862_2017_1002_MOESM6_ESM.pdf]

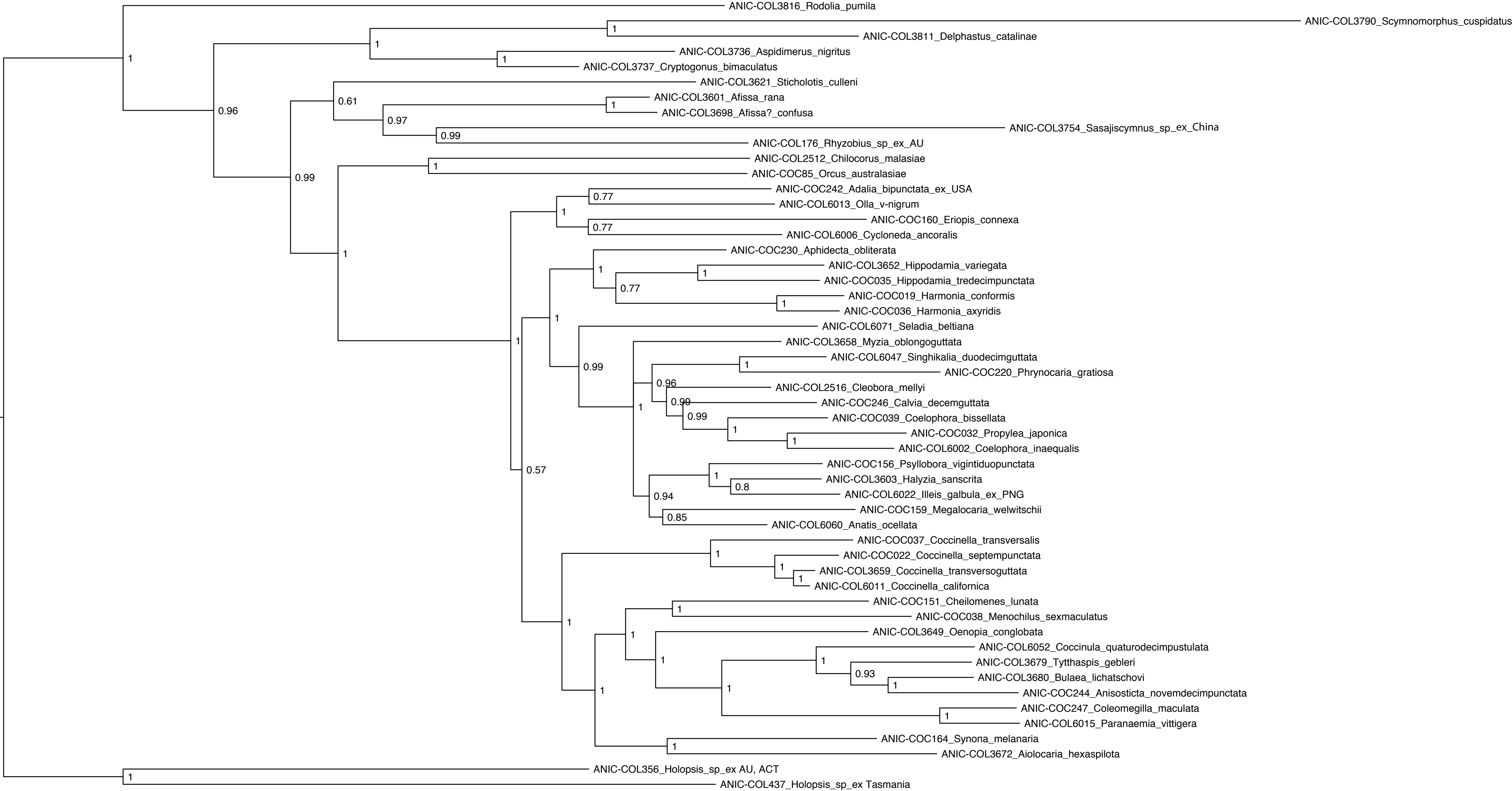

Supplement: Supplementary file 7 — The resulting tree of the MrBayes Bayesian analysis with posterior probabilities. (PDF 132 kb) [file 12862_2017_1002_MOESM7_ESM.pdf]

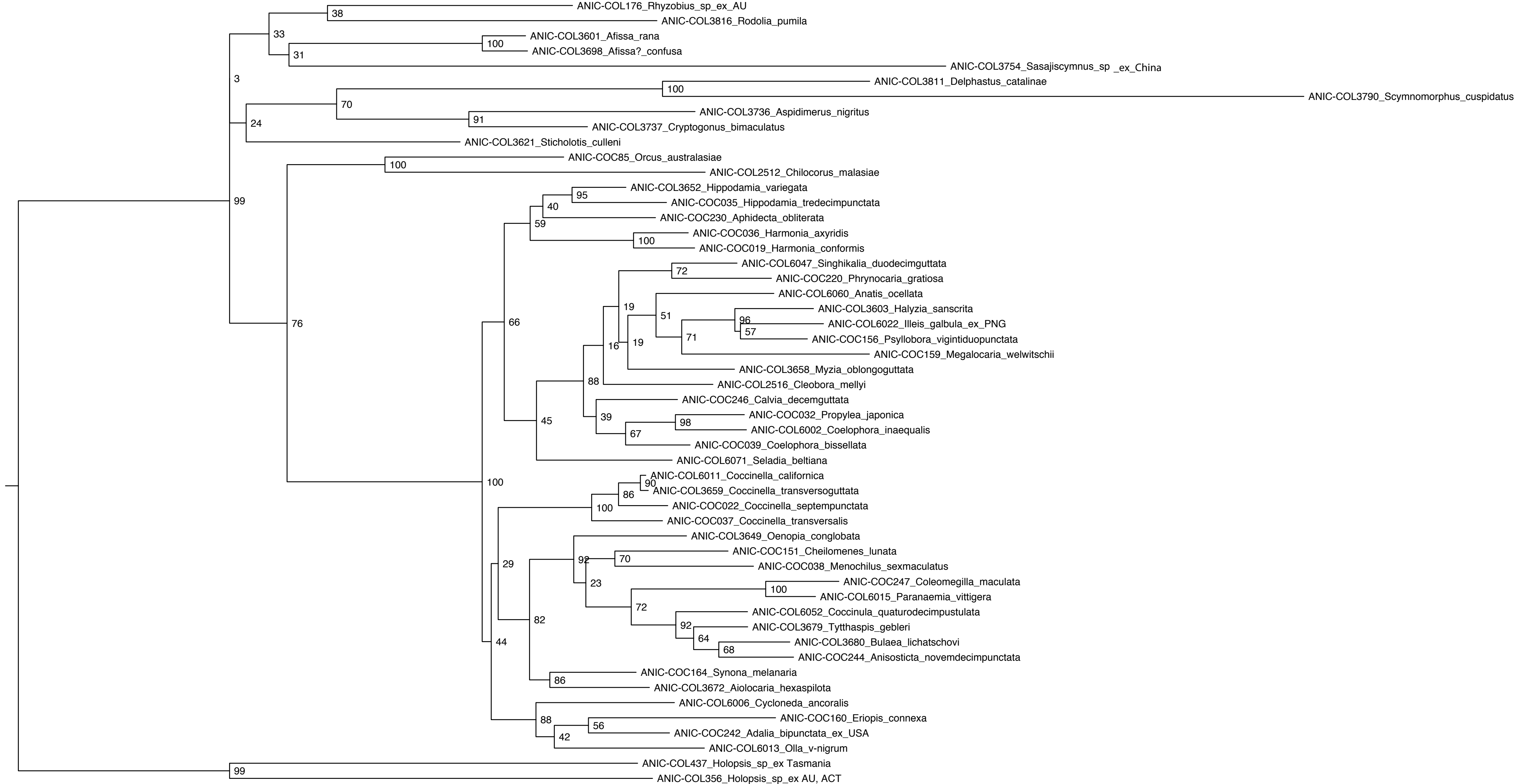

Supplement: Supplementary file 8 — The best RAxML ML tree with bootstrap values for the fully degenerated five-fragment MSA. (PDF 131 kb) [file 12862_2017_1002_MOESM8_ESM.pdf]

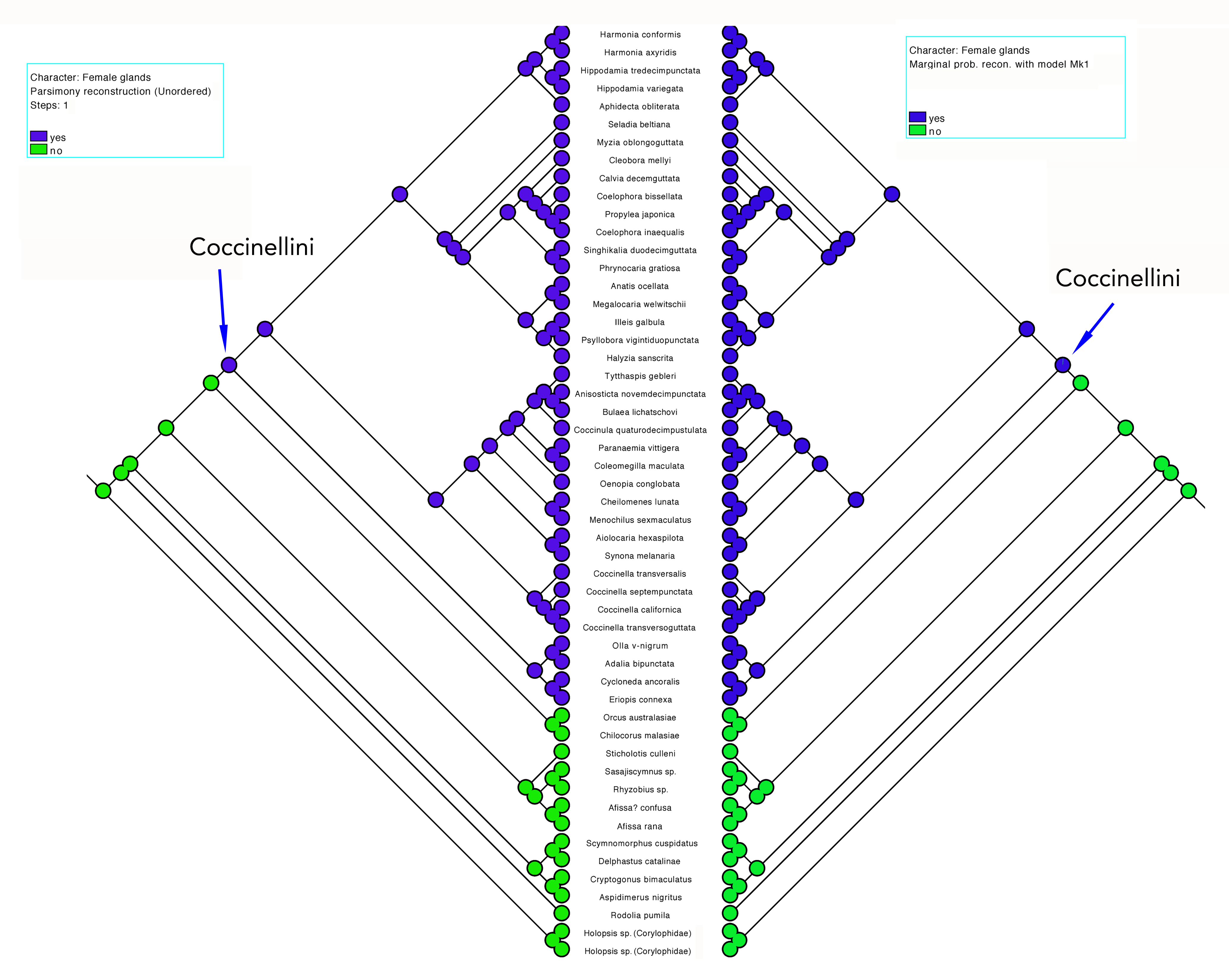

Supplement: Supplementary file 10 — Ancestral state reconstruction based on parsimony (A) and maximum likelihood (B) for female colleterial glands in Coccinellidae. The ancestral states are present (blue) and absent (green). The topology is derived from the ML tree in Fig. 3. (TIFF 39987 kb) [file 12862_2017_1002_MOESM10_ESM.tif]

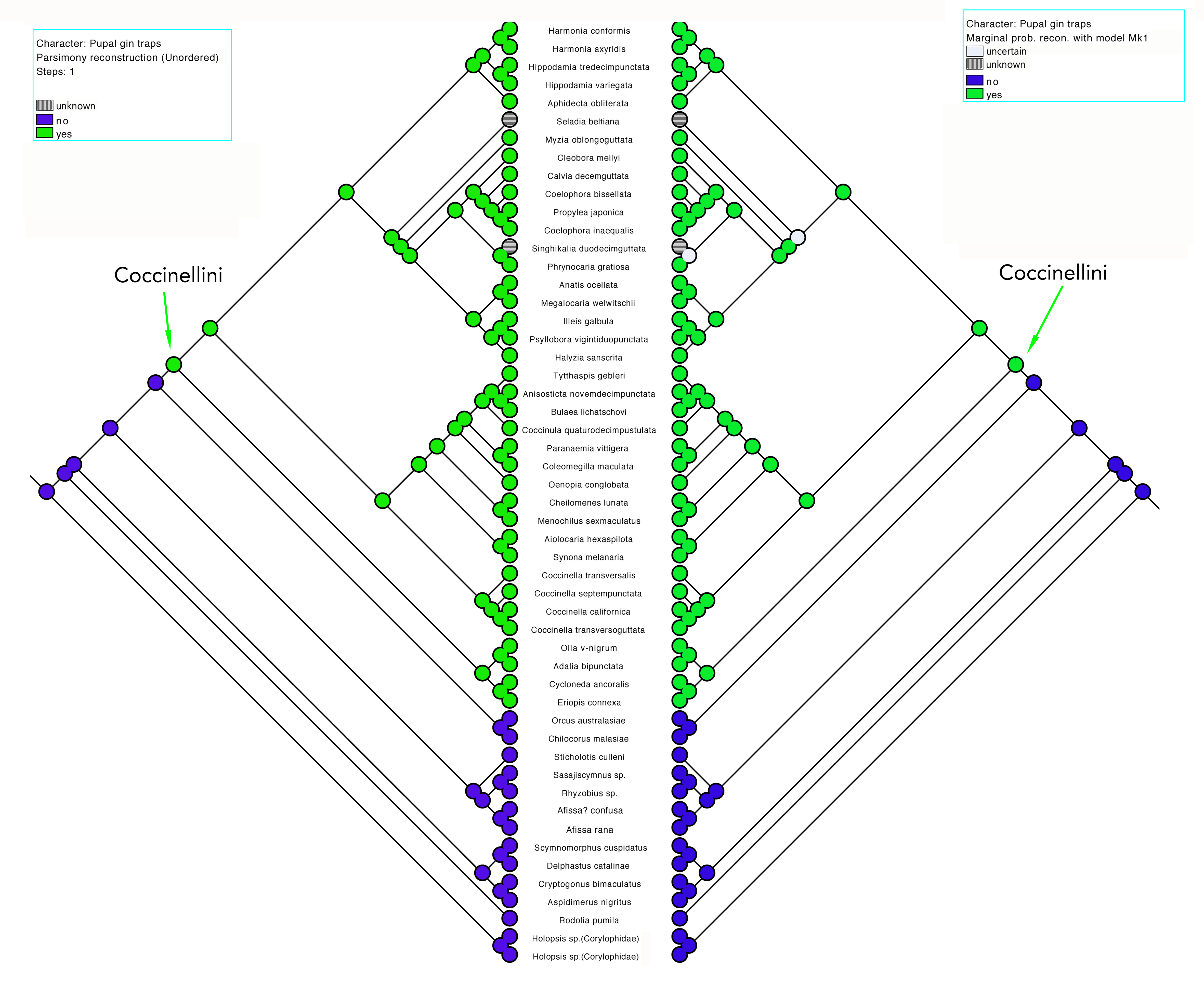

Supplement: Supplementary file 11 — Ancestral state reconstruction based on parsimony (A) and maximum likelihood (B) for pupal gin traps in Coccinellidae. The ancestral states are present (green), absent (blue), unknown (shadowed) and uncertain (grey). The topology is derived from the ML tree in Fig. 3. (TIFF 53439 kb) [file 12862_2017_1002_MOESM11_ESM.tif]

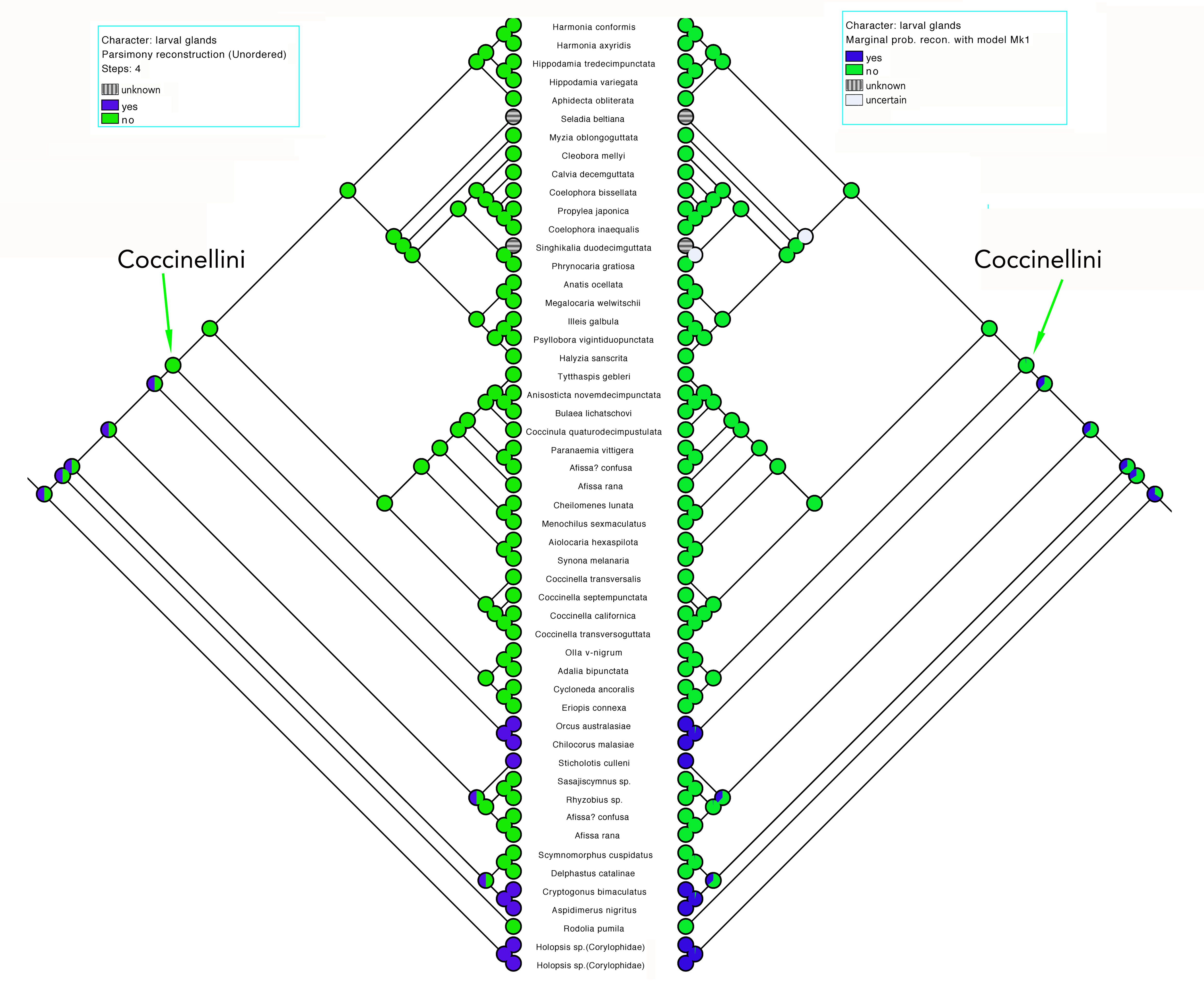

Supplement: Supplementary file 12 — Ancestral state reconstruction based on parsimony (A) and maximum likelihood (B) for larval dorsal glands in Coccinellidae. The ancestral states are present (blue), absent (green), unknown (shadowed) and uncertain (grey). The topology is derived from the ML tree in Fig. 3. (TIFF 60213 kb) [file 12862_2017_1002_MOESM12_ESM.tif]

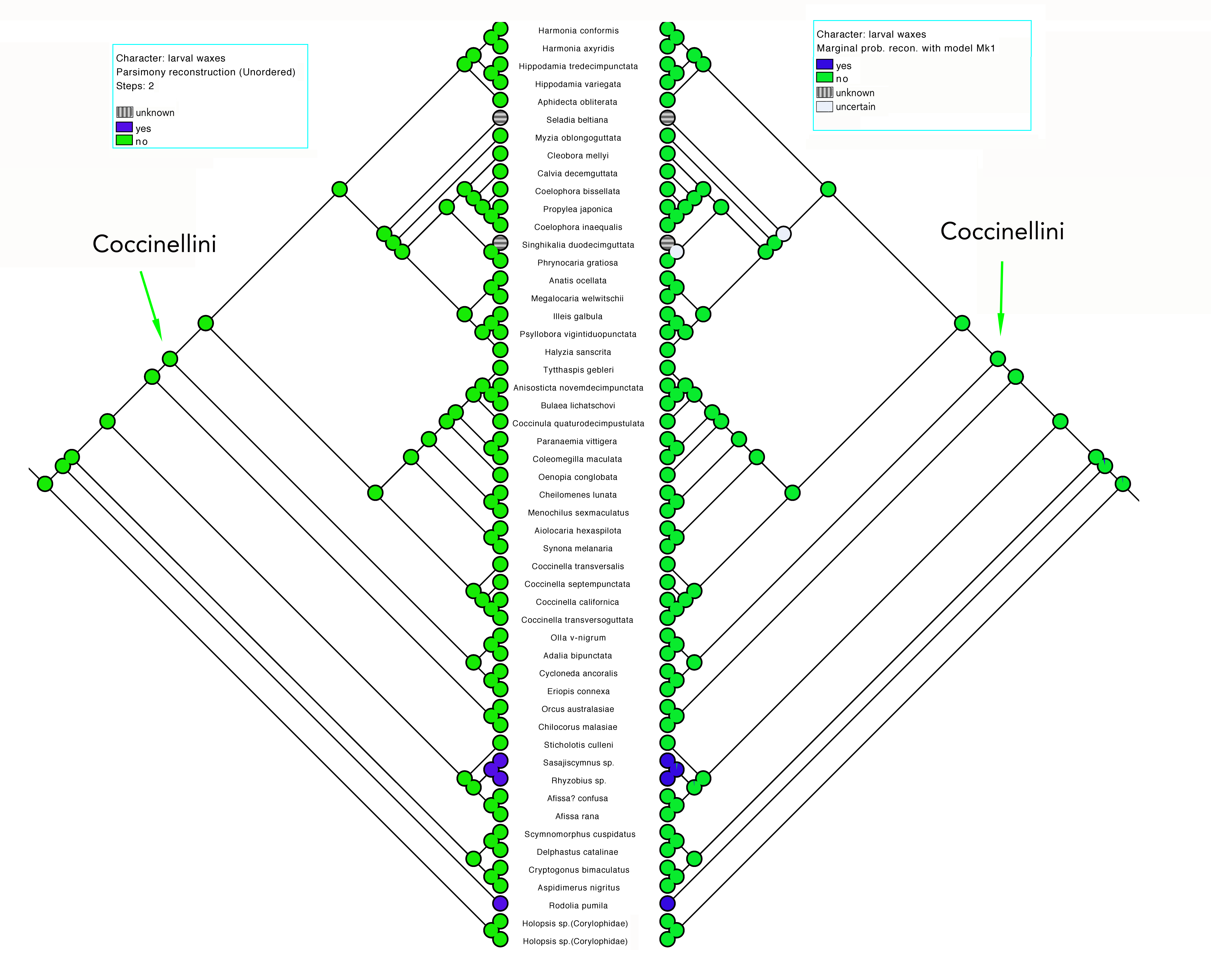

Supplement: Supplementary file 13 — Ancestral state reconstruction based on parsimony (A) and maximum likelihood (B) for larval waxes in Coccinellidae. The ancestral states are present (blue), absent (green), unknown (shadowed) and uncertain (grey). The topology is derived from the ML tree in Fig. 3. (TIFF 41014 kb) [file 12862_2017_1002_MOESM13_ESM.tif]

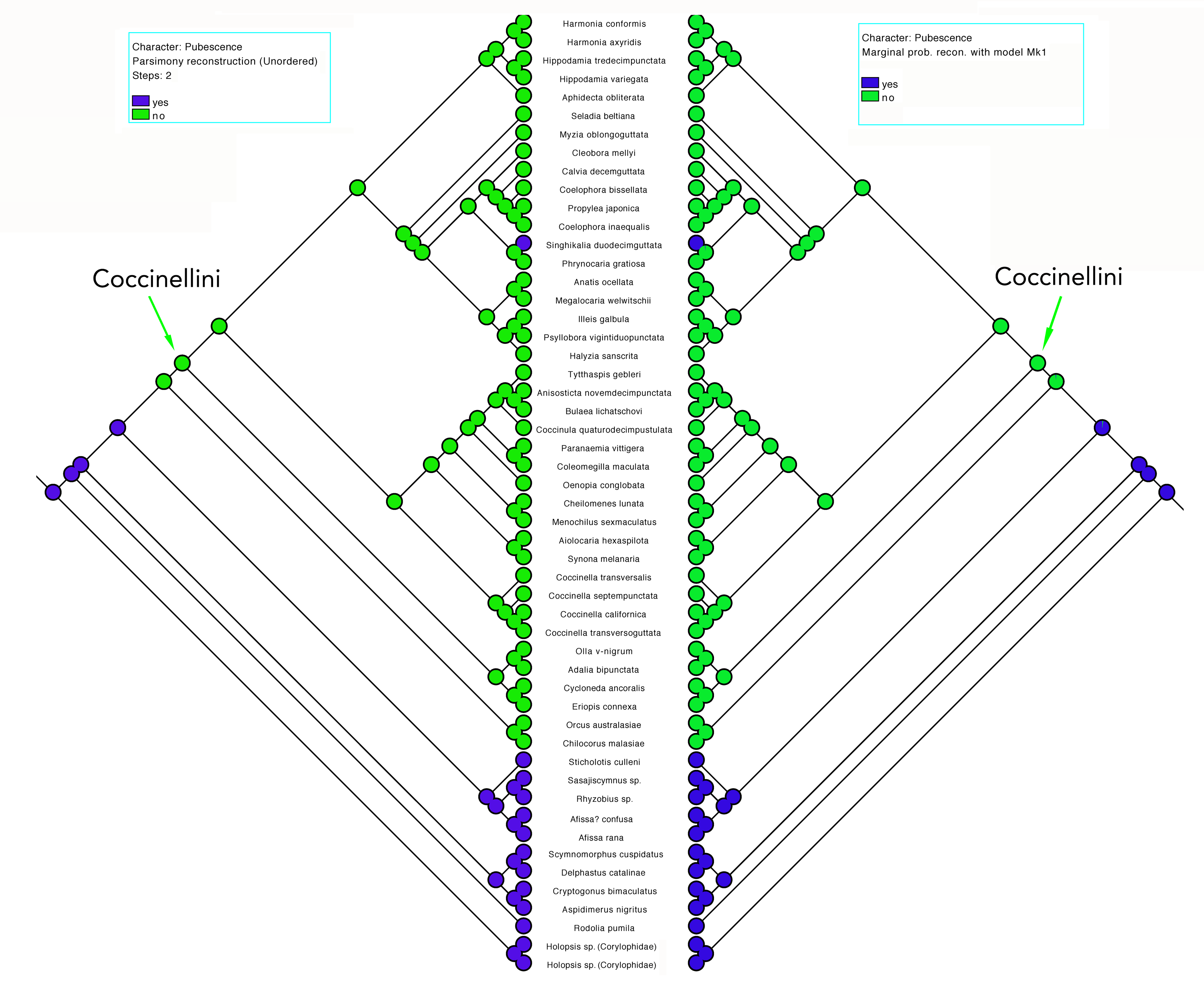

Supplement: Supplementary file 14 — Ancestral state reconstruction based on parsimony (A) and maximum likelihood (B) for presence of dorsal pubescence in Coccinellidae. The ancestral states are present (blue) and absent (green). The topology is derived from the ML tree in Fig. 3. (TIFF 59853 kb) [file 12862_2017_1002_MOESM14_ESM.tif]

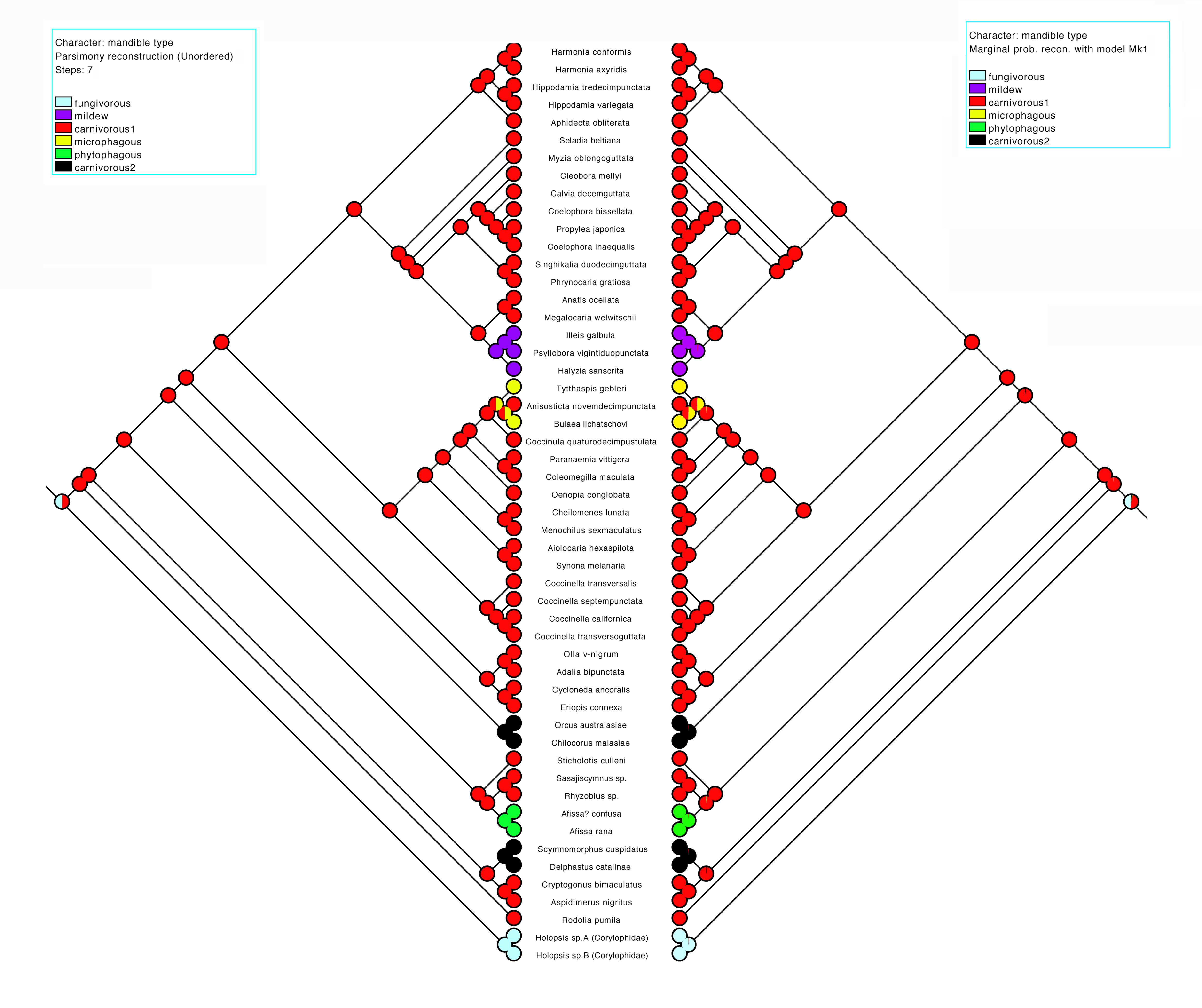

Supplement: Supplementary file 15 — Ancestral state reconstruction based on parsimony (A) and maximum likelihood (B) for mandible type in Coccinellidae. The ancestral states are separated on fungivorous (light blue), mildew (purple), carnivorous1 (red), microphagous (yellow), phytophagous (green), carnivorous2 (black). The topology is derived from the ML tree in Fig. 3. (TIFF 60492 kb) [file 12862_2017_1002_MOESM15_ESM.tif]

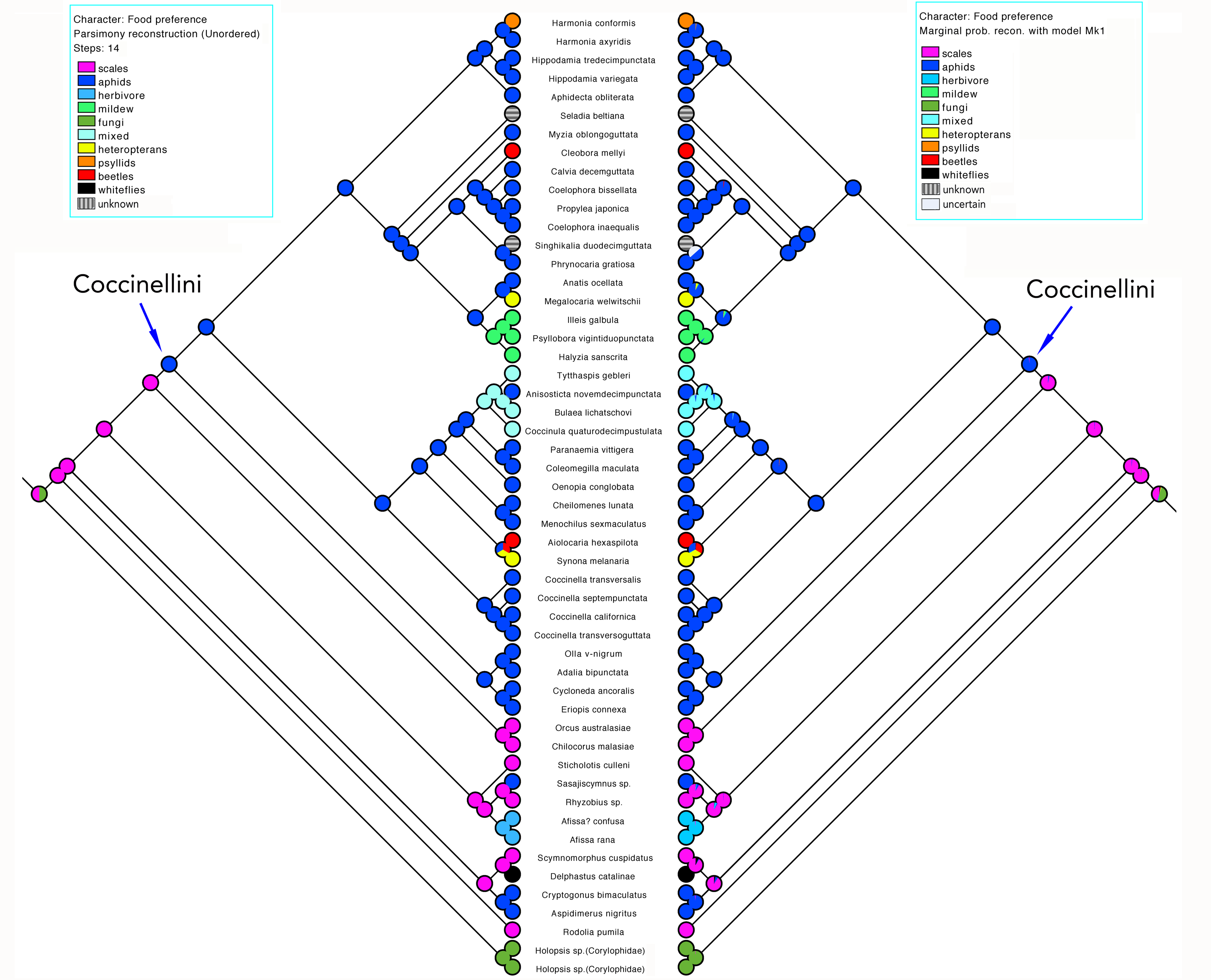

Supplement: Supplementary file 16 — Ancestral state reconstruction based on parsimony (A) and maximum likelihood (B) for food preferences in Coccinellidae. The ancestral states are separated on scales (purple), aphids (navy blue), herbivore (blue), mildew (light green), fungi (dark green), mixed (light blue), heteropterans (yellow), psyllids (orange), beetles (red), whiteflies (black), unknown (shadowed), uncertain (grey). The topology is derived from the ML tree in Fig. 3. (TIFF 58415 kb) [file 12862_2017_1002_MOESM16_ESM.tif]
